# Mechanochemical Generation of Active Pd/BippyPhos Catalyst for Efficient C—N Cross‐Coupling in Air

**DOI:** 10.1002/cssc.202500545

**Published:** 2025-04-21

**Authors:** Deniz Karabiyikli, Alexandre Saad, Sokaina Hammoud, Séverine Schneider, Romuald Manca, Jesus Raya, Martine Schmitt, Frédéric Bihel

**Affiliations:** ^1^ Laboratoire d’Innovation Thérapeutique UMR7200 CNRS Université de Strasbourg Faculté de pharmacie ‐ 74 route du Rhin 67401 Illkirch France; ^2^ Laboratory of Membranes Biophysics and NMR UMR7177 Institut de Chimie CNRS, Université de Strasbourg 4 rue Blaise Pascal 67070 Strasbourg France

**Keywords:** ball mill, cross‐coupling reaction, solid‐state NMR, solid‐state reactions, sustainable chemistry

## Abstract

Carbon—nitrogen (C—N) bond‐forming cross‐coupling reactions catalyzed by palladium‐based systems, known as Buchwald–Hartwig aminations, are widely used in natural product synthesis, pharmaceuticals, agrochemicals, and materials science. However, these reactions typically require organic solvents and inert atmospheres, such as argon, increasing environmental, health, and safety concerns. Using electron‐rich bulky phosphine ligands in combination with [Pd(π‐cinnamyl)Cl]_2_, a highly active palladium catalyst capable of achieving efficient C—N bond formation in the solid state is generated. Remarkably, while previous studies showed that the formation of this palladium–phosphine complex occurs only in protic solvents such as water or alcohols, but not in classical organic solvents, its generation in the absence of any solvent is demonstrated, as confirmed by solid‐state ^3^ ^1^P nuclear magnetic resonance, supporting its role as the active catalytic species. This process enables the coupling of a broad range of aryl bromides and chlorides with amines, anilines, amides, carbamates, or ureas, delivering good to excellent yields. This mechanochemical method operates with minimal palladium loading and proceeds efficiently in air, offering a practical and sustainable alternative to traditional solution‐phase reactions.

## Graphical abstract

A solvent‐free, solid‐state Buchwald–Hartwig amination is developed using [Pd(π‐cinnamyl)Cl]_2_ and bulky electron‐rich phosphines. The active catalyst forms without solvent or inert atmosphere, as confirmed by solid‐state ^3^ ^1^P nuclear magnetic resonance. This mechanochemical method enables efficient C—N bond formation with various nitrogen nucleophiles, offering a sustainable, low‐palladium, and air‐tolerant alternative to conventional solution‐phase protocols.

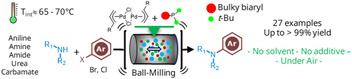

## 1 Introduction

The introduction of palladium as a catalyst for C(sp^2^)‐N bond formation can be traced back to 1983 when it was first explored by Migita et al.^[^ ^1^ ^]^ However, it was in the 1990s that this method gained significant attention and improvement, thanks to the independent work of Stephen L. Buchwald and John F. Hartwig.^[^ ^2^, ^3^, ^4^, ^5^ ^]^ Their advancements laid the foundation for what is now widely recognized as the Buchwald–Hartwig amination reaction.^[^ ^6^, ^7^ ^]^ This transformation has become a pivotal tool for synthetic chemists, particularly due to the importance of aryl amines and heterocycles, which are prevalent in a range of sectors including natural products, pharmaceuticals, agrochemicals, and materials science.^[^ ^8^, ^9^, ^10^, ^11^ ^]^ In a study by D. G. Brown and J. Boström, the Buchwald–Hartwig reaction was found to have been employed in roughly 10% of medicinal chemistry papers published in 2014, underscoring its widespread utility.^[^ ^11^ ^]^ In fact, it was highlighted as one of the top 20 most commonly utilized reactions within the pharmaceutical industry.^[^ ^11^, ^12^ ^]^ Over the past 30 years, significant progress has been made to optimize this reaction, particularly through the development of newer, more efficient catalysts and ligands.^[^ ^6^, ^7^, ^13^, ^14^, ^15^, ^16^, ^17^, ^18^, ^19^, ^20^, ^21^, ^22^, ^23^, ^24^, ^25^ ^]^ These advancements have enabled more streamlined and sustainable reaction conditions, driving continued innovation in this field. Traditionally, the Buchwald–Hartwig cross‐coupling reaction has been conducted in hydrocarbon‐based solvents, particularly toluene, or in cyclic ethers like tetrahydrofuran (THF) and 1,4‐dioxane.^[^ ^26^ ^]^ However, in the past decade, pharmaceutical companies such as Pfizer, GlaxoSmithKline (GSK), and Sanofi have issued guidelines to help medicinal chemists select solvents based on their environmental, health, and safety profiles.^[^ ^27^ ^]^ These guides generally deem toluene as “usable”, though Sanofi advises its replacement. THF is also considered “usable” by Pfizer but flagged as having “major issues” by GSK, while 1,4‐dioxane is universally categorized as “undesirable” by all three companies.

**Figure 1:**
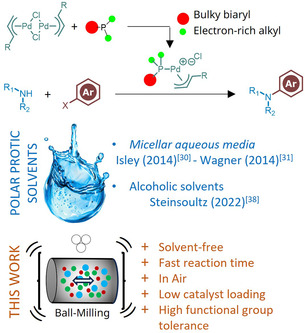
Buchwald–Hartwig cross‐coupling reaction using [Pd(π‐R‐allyl)Cl]_2_/Bulky and electron‐rich monophosphine catalyst system in green media.

In response to the need for safer alternatives, eco‐friendlier solvents have been proposed.^[^ ^27^ ^]^ In the field of Buchwald–Hartwig cross‐coupling reaction, green and sustainable nonpolar solvents, such as Me‐THF, MTBE, or TMO have emerged as viable alternatives to conventional organic solvents.^[^ ^28^, ^29^ ^]^ Polar protic solvents have also been investigated, and Lipshutz et al. reported a micellar Buchwald–Hartwig amination in aqueous media, employing TPGS‐750‐M as a surfactant in combination with the [Pd(π‐allyl)Cl]_2_/cBRIDP catalyst system.^[^ ^30^ ^]^ This approach has since been further optimized by several research groups, including our own.^[^ ^31^, ^32^, ^33^, ^34^, ^35^, ^36^ ^]^ Beyond water‐based systems, other polar protic solvents, such as ethanol (EtOH) and isopropanol (i‐PrOH), have also shown excellent efficiency in amination reactions.^[^ ^37^, ^38^ ^]^ Most studies utilizing these polar protic solvents have employed [Pd(π‐R‐allyl)Cl]_2_ in combination with bulky, electron‐rich monophosphine ligands (**Figure** **1**).^[^ ^30^, ^31^, ^32^, ^33^, ^34^, ^35^, ^36^, ^38^ ^]^ Colacot et al. have shown that in nonpolar organic solvents like THF or toluene, [Pd(π‐R‐allyl)Cl]_2_ cannot react with bulky phosphines, requiring prior treatment with silver triflate (AgOTf) to form the reactive cationic precatalyst, Pd(π‐R‐allyl)OTf, before introducing the monophosphine ligand.^[^ ^38^, ^39^ ^]^ Notably, this AgOTf treatment is unnecessary when polar protic solvents such as water or alcohols are used.^[^ ^30^, ^31^, ^32^, ^35^, ^36^, ^38^ ^]^ Steinsoultz et al. have recently characterized the in situ formation of the active cationic precatalyst Pd(π‐R‐allyl)Cl in alcohol‐based solvents (Figure 1).^[^ ^38^ ^]^

Although labeled as eco‐friendly, these solvents still generate waste at the end of the reaction, which must either be recycled or disposed of. Reducing or eliminating solvents is a crucial strategy for enhancing chemical processes, with solid‐state mechanochemistry leading the way toward solvent‐free synthesis. Mechanochemistry involves chemical reactions driven by mechanical forces, such as grinding or milling, and is a rapidly growing field, gaining recognition for its significant reduction or elimination of solvent use.^[^ ^40^, ^41^, ^42^, ^43^ ^]^ In the past decade, Pd‐catalyzed reactions under mechanochemical conditions have emerged as a promising yet relatively unexplored approach, offering new possibilities for sustainable catalysis.^[^ ^44^, ^45^, ^46^, ^47^, ^48^, ^49^, ^50^, ^51^, ^52^ ^]^ Despite the prominence of the Buchwald–Hartwig amination in organic and medicinal chemistry, mechanochemical approaches remain scarce, underscoring the need for further development in this area. Two catalytic systems were initially reported: Pd‐PEPPSI‐iPent demonstrated high efficiency for coupling secondary amines,^[^ ^53^ ^]^ while Pd(OAc)_2_, in combination with either XPhos or DavePhos, was employed for the coupling of N‐alkyl‐anilines and secondary amines, though it delivered moderate yields with primary amines or anilines.^[^ ^54^ ^]^ Recently, Genestre et al. reported a parallel mechanochemical optimization, demonstrating that the combined use of two palladium sources—Pd(OAc)_2_ and t‐BuXPhosPdG3—at a relatively high loading (6.5 mol% each), significantly improved yields compared to previous results achieved with Pd(OAc)_2_/DavePhos and Pd‐PEPPSI‐iPent.^[^ ^55^ ^]^ In 2020, Kubota et al. introduced the use of 1,5‐cyclooctadiene (1,5‐cod) with Pd(OAc)_2_, paired with either tri‐tert‐butylphosphine (*t*‐Bu_3_P) or the air‐stable tri(1‐adamantyl)phosphine (Ad_3_P).^[^ ^56^, ^57^, ^58^ ^]^ This approach enabled the efficient coupling of aniline derivatives, including those with high melting points. The same group published in 2022 the cross‐coupling reaction of carbazoles with aryl halides using Pd(OAc)2/*t*‐Bu_3_P.HBF_4_ under mechanochemical conditions at 125 °C.^[^ ^59^ ^]^

Given the proven efficacy of [Pd(π‐R‐allyl)Cl]_2_ with bulky, electron‐rich monophosphine ligands in the Buchwald–Hartwig reaction using polar protic solvents, and its inability to form the active catalytic species in conventional organic solvents such as toluene or THF, we investigated the activation potential of this precatalytic system under mechanochemical conditions—raising fundamental questions about the distinct reactivity enabled by solvent‐free, solid‐state environments.

## 2 Results and Discussion

Our study began by investigating the model coupling of 2‐bromonaphthalene (**1**) with 2‐amino‐4‐picoline (**2**) under ball mill conditions, using 5 mol% Pd catalyst and either sodium hydroxide or potassium tert‐butoxide as the base (**Table** **1**). All materials were weighed and added directly to the milling jar under ambient air, without any special precautions. The jars were then milled at 30 Hz for 90 min at room temperature. Under these conditions, the cross‐coupled product (**3a**) was obtained in a 31% yield using KOtBu (Table 1, entry 3), while no product was detected with NaOH (Table 1, entry 1). By heating the reactors during the milling process using heat guns positioned above them (preset to 100 °C, see the setup in Section S2, Supporting Information), the KOtBu‐mediated reaction yielded compound **3a** with 71% (Table 1, entry 7), whereas only 6% yield was obtained with NaOH (Table 1, entry 2). Using a thermographic camera to measure the internal reactor temperature (*T*_int_ as the highest hot spot) after opening, we recorded a *T*_int_ of 38 °C after 90 min at room temperature, compared to 69 °C after 90 min with heat gun set to 100 °C. Increasing the external temperature (*T*_ext_) to 50, 60, and 80 °C resulted in a corresponding rise in yields (Table 1, entries 4–6), with a plateau observed above 80 °C (*T*_ext_). We then monitored the internal temperature of the ball‐mill reactor over the 90‐min reaction period with a *T*_ext_ of 100 °C. Using the thermographic camera at various time points, we found that the internal temperature reached a plateau within ≈10 min (Section S2, Figure S3, Supporting Information), indicating that the reaction proceeds under stable temperature conditions for the majority of the reaction time.

**Table 1:** Proof of concept of a Buchwald–Hartwig cross‐coupling reaction using [Pd(π‐allyl)Cl]_2_/*t‐*.BuXPhos under mechanochemical conditions.

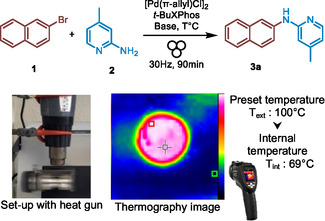				
Entrya)	Bases	*T*_ext_ b) [°C]	*T*_int_ c) [°C]	Yieldd) [%]
1	NaOH	RT	38	0
2	NaOH	100	69	6
3	KOtBu	RT	38	31
4	KOtBu	50	51	55
5	KOtBu	60	54	65
6	KOtBu	80	62	72
7	KOtBu	100	69	71

a) Conditions: ArBr **1** (0.22 mmol), RNH_2_ **2** (0.33 mmol), [Pd(π‐allyl)Cl]_2_ (5 mol%), *t‐*BuXPhos (10 mol%), NaOH or KOtBu (0.33 mmol) in a stainless‐steel ball‐milling jar (5 mL) with 3 stainless‐steel ball (*d* = 10 mm).

b) A heat gun was used to heat the jar (*T*_ext_).

c) The temperature (*T*_int_) inside the milling jar after the solid‐state coupling reaction was measured by thermography. *T*_int_ is the average of at least 2 reactions.

d) Yields are the average of at least two reaction, and were determined using ^1^H NMR with caffeine as an external standard.

We then screened a series of monophosphines as ligands in combination with [Pd(π‐allyl)Cl]_2_ in the presence of KOtBu with a *T*_ext_ = 100 °C for 90 min at 30 Hz. While phosphines bearing cyclohexyl groups resulted in no reaction (**Table** **2**, entries 1 and 2), electron‐rich monophosphines with *t*‐Bu moieties successfully produced the desired compound **3a** in good to excellent yields, ranging from 53% to 88%, with the exception of Me_4_‐t‐BuXPhos, which gave only a 15% yield (Table 2, entry 9). While the electron‐rich nature of the monophosphine ligand proved essential for the catalytic process, its bulkiness seemed less critical. This is evident from JohnPhos, which has an unsubstituted biaryl group and still achieved a 63% yield. BippyPhos and RockPhos emerged as the most promising ligands, but due to the high cost of RockPhos, we opted to continue the study using BippyPhos. Building on this result, we refined the reaction parameters to further optimize the outcome, starting with the choice of base (**Table** **3**, entries 1–9) in presence of [Pd(π‐allyl)Cl]_2_ (3 mol%) and BippyPhos (6 mol%). Various alkali metal alkoxides, analogous to KOtBu, were screened, including potassium, sodium, and lithium in combination with primary, secondary, or tertiary alkoxides. Most bases provided yields between 64% and 73%, except for LiOtBu, which only achieved a 37% yield (Table 3, entries 1–7). In contrast, K_2_CO_3_ and K_3_PO_4_ resulted in significantly lower yields of 20% and 40%, respectively (Table 3, entries 8–9). Next, we examined the effect of both the number and diameter of the milling balls (Table 3, entries 10–14). The resulting yields were fairly consistent, ranging from 56% to 70%, with 10 mm beads showing a slight advantage.

**Table 2:** Screening of ligands.

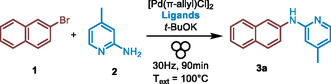		
Entrya)	Ligands	Yield [%]b)
1	CyJohnPhos	0
2	XPhos	0
3	JohnPhos	63
4	*t*‐BuDavePhos	53
5	*t*‐BuXPhos	69
6	BippyPhos	88
7	RockPhos	88
8	*t*‐BuBrettPhos	79
9	Me_4_‐*t*‐BuXPhos	15

a) Conditions: ArBr **1** (0.22 mmol), RNH_2_ **2** (0.33 mmol), [Pd(π‐allyl)Cl]_2_ (5 mol%), *t‐*BuXPhos (10 mol%), KOtBu (0.33 mmol) in a stainless‐steel ball‐milling jar (5 mL) with 3 stainless‐steel ball (*d* = 10 mm).

b) Yields are the average of at least two reaction, and were determined using ^1^H NMR with caffeine as an external standard.

**Table 3:** Screening of bases and beads.

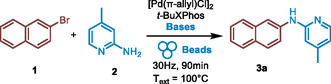				
Entrya)	Bases	Stainless‐steel balls		Yield [%]b)
		Number	diameter [mm]	
1	KOtBu	3	10	69
2	NaOtBu	3	10	64
3	LiOtBu	3	10	37
4	NaOEt	3	10	73
5	KOEt	3	10	71
6	NaOMe	3	10	66
7	KOMe	3	10	64
8	K_2_CO_3_	3	10	20
9	K_3_PO_4_	3	10	40
10	KOtBu	4	10	70
11	KOtBu	2	10	64
12	KOtBu	1	10	64
13	KOtBu	3	7	61
14	KOtBu	3	5	56


a) Conditions: ArBr **1** (0.22 mmol), RNH_2_ **2** (0.33 mmol), [Pd(π‐allyl)Cl]_2_ (3 mol%), *t‐*BuXPhos (6 mol%), base (0.33 mmol) in a stainless‐steel ball‐milling jar (5 mL).

b) Yields are the average of at least two reaction, and were determined using ^1^H NMR with caffeine as an external standard.

As a final step in the optimization process, we evaluated the influence of the π‐R‐allyl group on reactivity with the aim of reducing the palladium loading. Previous studies by Melvin et al. have demonstrated that the nature of the allylic group can significantly affect catalytic performance.^[^ ^60^ ^]^ Consequently, we tested [Pd(π‐allyl)Cl]_2_, [Pd(π‐crotyl)Cl]_2_, and [Pd(π‐cinnamyl)Cl]_2_ at varying loadings, using BippyPhos as the ligand and either EtONa or KOtBu as the base (**Table** **4**). EtONa proved to be a superior base compared to KOtBu, which exhibited limited efficiency. For instance, KOtBu gave suboptimal results, yielding only 64% with 3 mol% of [Pd(π‐cinnamyl)Cl]_2_ (Table 4, entry 1) and just 46% and 21% with 0.5 mol% of [Pd(π‐allyl)Cl]_2_ and [Pd(π‐crotyl)Cl]_2_, respectively (Table 4, entry 3). In contrast, NaOEt delivered excellent yields across all three palladium sources down to a loading of 0.5 mol% (Table 4, entries 1‐3), although yields diminished to 40–50% at a reduced loading of 0.25 mol% (Table 4, entry 4). We hypothesized that reducing the catalyst loading might require longer reaction times. Therefore, we tested a 3‐hour reaction at 100 °C using both [Pd(π‐crotyl)Cl]_2_ and [Pd(π‐cinnamyl)Cl]_2_ at a 0.25 mol% catalyst loading (Table 4, entry 5). [Pd(π‐crotyl)Cl]_2_ showed a significant improvement, achieving a 73% yield, whereas [Pd(π‐cinnamyl)Cl]_2_ produced a yield comparable to that observed after 90 min. These results suggest that [Pd(π‐crotyl)Cl]_2_ may offer a slight advantage over its cinnamyl counterpart. However, it is important to note that [Pd(π‐crotyl)Cl]_2_ is significantly more expensive than the other [Pd(π‐allyl)Cl]_2_ or [Pd(π‐cinnamyl)Cl]_2_ complexes.

**Table 4:** Palladium sources and loading.

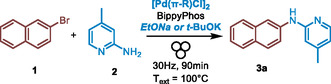							
		Yield [%]b)					
[Pd(π‐R)Cl]_2_ :a)		Allyl		Crotyl		Cinnamyl	
Entry	Pd [mol%]	NaOEt	KOtBu	NaOEt	KOtBu	NaOEt	KOtBu
1	3	90	87	91	91	95	64
2	1	88	90	89	88	93	nd
3	0.5	87	46	90	21	86	nd
4	0.25	45	ndc)	52	0	42	nd
5d)	0.25	nd	nd	73	0	44	nd

a) Conditions: ArBr **1** (0.22 mmol), RNH_2_ **2** (0.33 mmol), [Pd(π‐allyl)Cl]_2_ (3 mol%), *t‐*BuXPhos (6 mol%), base (0.33 mmol) in a stainless‐steel ball‐milling jar (5 mL).

b) Yields were determined using ^1^H NMR with caffeine as an external standard.

c) nd: not determined.

d) modification of the standard conditions: Upscaling of the reaction with ArBr **1** (0.44 mmol, 1 eq), for a reaction time of 3 h.

With the optimal conditions established, we explored the reaction scope using a range of aryl halides (including aryl and heteroaryl chlorides or bromides) in combination with various NH‐containing reactants, such as aryl/heteroarylamines, primary and secondary amines, amides, carbamates, and ureas (**Table** **5**). All reactions were carried out under ambient air conditions, and [Pd(π‐cinnamyl)Cl]_2_ being preferred due to cost considerations. While previous optimization steps utilized 1.5 equivalents of amine per 1 equivalent of ArX, we observed that reducing the amine to 1.1 equivalents resulted in comparable yields. Consequently, this ratio was applied in the scope of the reaction. First, a series of aryl halides were coupled with 2‐amino‐picoline. Various bromo‐ and chloro‐substituted benzene and naphthalene rings were efficiently coupled, yielding excellent to nearly quantitative results (**3b**–**d**, **3i**–**j**). In the case of heteroaryl halides, 3‐chloropyridine coupled with 2‐amino‐picoline in a quantitative yield (**3h**), while 2‐bromoquinoline gave a yield of 38% (**3p**), and 2‐bromo‐thiophene or 8‐bromoquinoline produced only trace amounts of the coupling products (**3n** and **3o**, respectively). For these three examples, we hypothesize that reaction poisoning may have occurred due to the formation of a “pincer” in the coupling product, trapping the palladium catalyst. Next, 2‐bromonaphthalene was coupled with a range of mono‐alkylated anilines (**3e**–**g**, **3k**–**m**, **3q**–**r**) and di‐alkylated aniline (**3s**), resulting in yields from 64% to nearly quantitative. Primary and secondary amines were also efficiently coupled with 2‐bromonaphthalene (**3t**–**y**), although 1‐adamantamine (**3v**) produced a lower yield of 46%, likely due to steric hindrance from the bulky adamantane group. While 4‐methoxybenzamide was efficiently coupled with 2‐bromonaphthalene, producing an excellent yield of 90% (**3ac**), benzamide unexpectedly resulted in a poor yield of 33% (**3ab**) under standard conditions. However, the yield was modestly improved to 65% when the Pd catalyst loading was increased to 3 mol%. Eventually, the carbamate BocNH_2_ afforded a modest yield of 34% (**3z**), while N,N‐dimethylurea provided a more convenient yield of 73% for product **3aa**.

**Table 5:** Scope of the reaction.a)

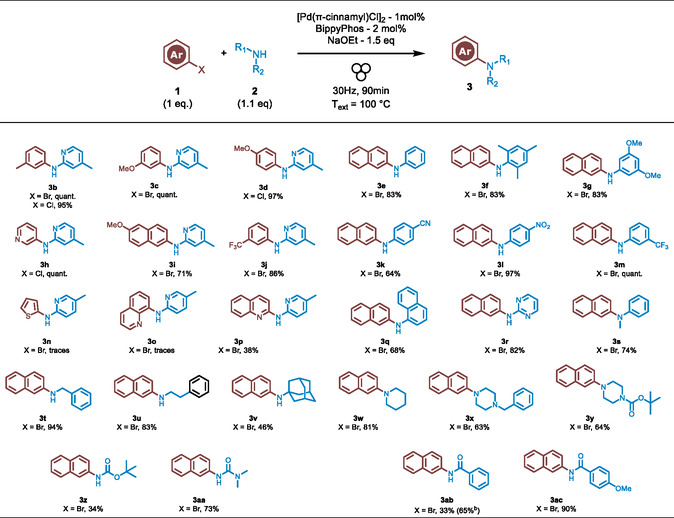

a) Reaction conditions: ArBr **1** (0.22 mmol), RNH_2_ **2** (0.24 mmol), [Pd(π‐cinnamyl)Cl]_2_ (1 mol%), *t‐*BuXPhos (2 mol%), base (0.33 mmol) in a stainless‐steel ball‐milling jar (5 mL) with 3 stainless‐steel ball (*d* = 10 mm).

**Figure 2:**
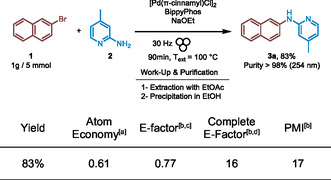
Green metrics applied to gram‐scale synthesis of **3a.** (a) Conditions: 2‐bromonaphthalene (1 eq., 1035 mg, 5 mmol), 2‐amino‐4‐picoline (1.1 eq., 595 mg, 5.5 mmol), Bippyphos (0.02 eq., 50.7 mg, 0.1 mmol), [Pd(cinnamyl)Cl]_2_ (0.01 eq., 25.9 mg, 0.05 mmol) and EtONa (1.5 eq., 510.4 mg, 7.5 mmol) in a stainless‐steel ball‐milling jar (10 mL). (b) Formula are described in SI. (c) E‐factor calculated by neglecting the quantities of solvents used for work‐up and purification. (d) Complete E‐factor including work‐up and purification.

Using the model reaction, a scaled up was performed using up to 5 mmol of 2‐Bromonapthalene and 5.5 mmol of 2‐amino‐4‐picoline (**Figure** **2**). The reaction was performed at 30 Hz in a 10 mL ball‐milling jar with 3 grinding balls (10 mm diameter) for 90 min at 100 °C (*T* ext). The solids were recovered with a minimum of EtOAc, filtered, and after evaporation of the volatiles, the resulting crude was precipitated in EtOH to give the expected product **3a** in 83% yield and an HPLC purity above 98% (254 nm). Green metrics were determined for this gram‐scale reaction (Figure 2).^[^ ^61^, ^62^ ^]^ As expected for a mechanochemical process, the E‐Factor, calculated without considering solvents used for work‐up and purification, was remarkably low (E‐Factor = 0.77). Notably, the straightforward work‐up and simple product precipitation in EtOH minimized solvent usage, resulting in a complete E‐Factor of 16.

In a previous study using [Pd(π‐allyl)Cl]_2_ with t‐BuXPhos as the ligand, we demonstrated the formation of a cationic monomeric palladium precatalyst ([*t‐*BuXPhosPd(π‐allyl)]Cl) in the presence of alcoholic solvents (Figure 1),^[^ ^38^ ^]^ while the same reaction did not occur in organic solvents such as THF or toluene.^[^ ^38^, ^39^ ^]^ Since we have shown that the combination of [Pd(π‐allyl)Cl]_2_ with bulky, electron‐rich monophosphines enables the Buchwald–Hartwig coupling reaction under mechanochemical conditions, it is reasonable to hypothesize that a similar precatalyst could form in a ball mill reactor. However, without using solvents, it is challenging to confirm this hypothesis using traditional analytical techniques.

**Figure 3:**
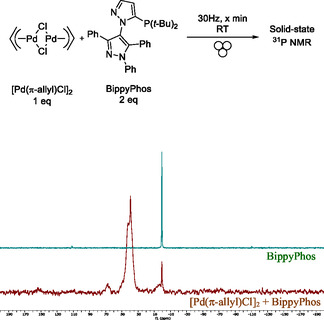
Magic angle spinning (MAS) solid‐state ^31^P NMR of the crude solid resulting from the reaction between [Pd(π‐cinnamyl)Cl]_2_ and BippyPhos in a ball‐mill. Conditions: [Pd(π‐allyl)Cl]2 (0.041 mmol), BippyPhos (0.082 mmol) in a stainless‐steel ball‐milling jar (5 mL) with 1 stainless‐steel bead (10 mm) for 30 min at 30 Hz at RT. The resulting crude mixture was transferred in air to solid‐state NMR rotor (3.2 mm) and studied by ^31^P NMR (top) in comparison with solid BippyPhos alone (bottom) which was separately grinded at RT for 5 min at RT. NMR conditions: ^31^P CP (cross‐polarization), contact time 3 ms, field 500 MHz, MAS frequency 22.5 kHz, temperature 303 K.

To address this issue, solid‐state nuclear magnetic resonance (NMR) was described as adequate spectroscopic approach.^[^ ^63^ ^]^ Thanks to the use of monophosphine as ligand, we employed solid‐state ^31^P NMR to detect potential changes in the phosphine signal of BippyPhos. As shown in **Figure** **3**, the ^31^P NMR signal of BippyPhos shifted from 0.6 ppm (BippyPhos alone) to a broad signal ranging from 30 to 53 ppm after ball milling with [Pd(π‐allyl)Cl]_2_. The presence of a residual peak corresponding to free BippyPhos suggests that the new signal arises from the complexation between [Pd(π‐allyl)Cl]_2_ and BippyPhos. While these data support the formation of a palladium complex, solid‐state ^31^P NMR cannot definitively determine the structure or confirm the formation of a cationic monomeric precatalyst like that observed in alcoholic solvents. The crude product from the grinding of Pd‐allyl and BippyPhos, analyzed by solid‐state ^31^P NMR, was used the following day in a cross‐coupling reaction between 2‐bromonaphthalene and 2‐amino‐picoline, successfully yielding the desired product (**3a**). This result demonstrates that the palladium complex formed via mechanochemistry is active and remains stable for at least 24 h when exposed to air at room temperature.

## 3 Conclusion

In summary, we have disclosed an efficient catalytic system for mechanochemical aromatic amination. Notably, this system operates efficiently with a low catalyst loading (≤1 mol%), which, given the dimeric nature of the precatalyst ([Pd(π‐R‐allyl)Cl]_2_), corresponds to less than 2 mol% of palladium. It accommodates a wide substrate scope, including the coupling of chloro‐ and bromo‐aryl compounds with a variety of partners such as anilines (both N‐alkylated and nonalkylated), primary and secondary amines, amides, carbamates, and ureas. Additionally, the observed internal temperature of ≈70 °C in the reactor suggests the possibility of a liquid‐phase reaction, potentially triggered by the melting of reactants. While some reactants within the scope have relatively low melting points (<70 °C), this is not true for all cases, such as the coupling of 2‐bromo‐6‐methoxynaphthalene (mp = 106–109 °C) with 2‐amino‐picoline (mp = 96–99 °C). Furthermore, all reactions consistently yielded a paste‐like consistency, a typical observation in mechanochemistry.

The good to excellent yields achieved across the scope also negated the need for liquid‐assisted grinding or additives, simplifying the recovery of crude products through straightforward dissolution in an appropriate solvent. Using solid‐state ^31^P NMR, we successfully demonstrated the mechanochemically mediated formation of a palladium complex without the need for any base or additives, mirroring results previously observed in alcoholic solvents. While the combination of [Pd(π‐R‐allyl)Cl]_2_ and bulky electron‐rich monophosphine ligands has already been identified as a practical catalytic system in polar protic solvents such as water or alcohols, this work extends its applicability to solvent‐free mechanochemical conditions. This provides an eco‐friendly alternative to conventional palladium‐catalyzed C—N bond‐forming reactions in organic solvents, offering a greener route for the synthesis of valuable complex molecules.

## Conflict of Interest

The authors declare no conflict of interest.

## Author Contributions

**Deniz Karabiyikli**: methodology, investigation, visualization; **Alexandre Saad**: investigation; **Sokaina Hammoud**: investigation; **Séverine Schneider**: investigation; **Romuald Manca**: investigation; **Jesus Raya**: methodology, investigation; **Martine Schmitt**: conceptualization, methodology, writing—original draft, supervision; **Frédéric Bihel**: conceptualization, methodology, writing—original draft, visualization, supervision, project administration. All authors have given approval to the final version of the manuscript.

## Supporting information

Supplementary Material

## Data Availability

The data that support the findings of this study are available in the supplementary material of this article.
