# Supplementary material for: Mechanochemical Generation of Active Pd/BippyPhos Catalyst for Efficient C—N Cross‐Coupling in Air
Source: ChemSusChem. 2025 Apr 21;18(13):e202500545. doi: 10.1002/cssc.202500545 (PMC12232107; doi:10.1002/cssc.202500545)

Supporting Information

Mechanochemical Generation of Active Pd/BippyPhos Catalyst for Efficient C─N Cross-Coupling in Air

*D. Karabiyikli,[1] A. Saad,[2] S. Hammoud,[1] S. Schneider,[1] R. Mancua,[2] J. Raya,[2] M. Schmitt,[1] F. Bihel[1]**

[1] Laboratoire d'Innovation Thérapeutique, UMR7200, CNRS, Université de Strasbourg, 67401 Illkirch, France.

[2] Univ Strasbourg, Institut de Chimie,, CNRS, UMR 7177, 4 Rue Blaise Pascal, 67008 Strasbourg, France

Email : fbihel@unistra.fr

Table of Contents

SI-§1. Materials and Instruments 2
SI-§2. Set-Up Procedure for High-Temperature Ball-Milling Reactions 3
SI-§3. General Procedure for Solid-State C–N Coupling Using a Ball Mill. 5
SI-§4. Procedure for Solid-State C–N Coupling on a Gram Scale. 6
SI-§5. Solid-state NMR analysis 7
SI-§6. Characterization of the compounds 3a-ac 9
SI-§7. Bibliography 19
SI-§8. NMR Traces 21

SI-§1. Materials and Instruments

All commercial reagents were used without purification. Analytical TLC was performed using Merck 60 F254 silica gel plates, and visualized by exposure to ultraviolet light (254 nm). Compounds were purified on silica gel Merck 60 (particle size 0.040−0.063 nm). NMR spectra were recorded on Bruker Avance III spectrometers. Operating at 500, 400, or 300 MHz for 1H, 126, 101, or 75 MHz for 13C, 202 or 121 MHz for 31P and 470 or 282 MHz for 19F. The 31P NMR spectra were recorded at 121 MHz. All chemical shift values δ and coupling constants J are quoted in ppm and in Hz, respectively, multiplicity (s= singlet, d= doublet, t= triplet, q= quartet, m= multiplet, and br = broad). Analytical RP-HPLC-MS was performed using a LC 1200 Agilent with quadrupole-time-of-flight (QTOF) (Agilent Accurate Mass QToF 6520) with a ZORBAX Agilent C18-column (C18, 50 mm × 2.1 mm; 1.8 μm) using the following parameters: (1) the solvent system: A (0.05% of formic acid in acetonitrile) and B (0.05% of formic acid in H2O); (2) a linear gradient: t = 0 min, 98% B; t = 8 min, 0% B; t = 12.5 min, 0% B; t = 12.6 min, 98% B; t = 13 min, 98% B; (3) flow rate of 0.5 mL/min; (4) column temperature: 35 °C; (5) DAD scan from 190 to 700 nm; and (6) ionization mode: ESI+. HPLC were performed using a Dionex UltiMate 3000 using the following parameters: column temperature: 40°C, from t = 0 min to t = 3.80 min the flow rate goes from 0.650 mL/min to 0.900 ml/min with the solvent system: A (0.05% of TFA in H2O) and B (MeCN) with 5 to 100% of B, then after t = 3.80 min to the end of the run t = 5.50 min: 100% of B at 0.900 ml/min. All mechanochemical reactions were carried out using grinding vessels in a Retsch MM400 mill jars (1.5 mL, 5 mL or 10 mL) and balls are made of stainless. The heat gun Steinel HG2310 LDC and Steinel HG2320 E with temperature control function was used for the high-temperature ball-milling reactions. Thermography was measured with the Thermal Imager PCE-TC 28 from PCE-instruments.

SI-§2. Set-Up Procedure for High-Temperature Ball-Milling Reactions

1. *Set-up procedure*.

A heat gun was fixed with clamps and placed directly above the ball milling jar (Figure S1). The distance between the heat gun tip and the ball milling jar was determined by thermographic analysis in order to ensure consistent and reproducible temperatures inside the jar during the reaction (see next section).

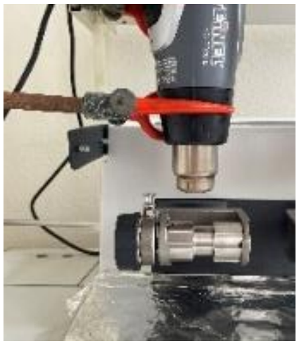

Fig. S1 : The set-up procedure for a heat gun on MM400

2. *Thermographic Analysis*.

The temperature inside the milling jar was measured for each reaction using thermography immediately upon opening. (Fig. S2)

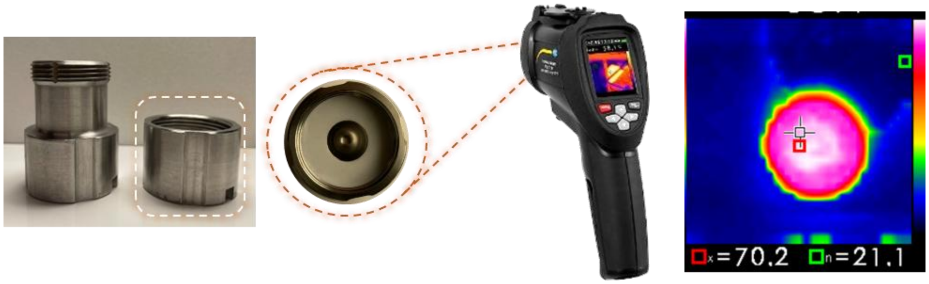

Fig. S2 : Thermography image of the reaction mixtures after ball milling at 30 Hz for 90 min with a heat gun set to an external temperature (T_{ext}) of 100°C.

The distance between the ball milling jar and the heat gun was adjusted to achieve an internal jar temperature (T_{int}) of 75 ± 3 °C after 90 min of heating, with the heat gun pre-set to 100°C (Text) and without grinding balls.

3. Evolution of the internal temperature of the jar over time.

The distance between the ball milling jar and the heat gun was adjusted to achieve an internal jar temperature (Tint) of 75 ± 3°C after 90 minutes of heating, with the heat gun set to 100°C (T_{ext}) and without grinding balls. We then measured the internal temperature (T_{int}) of the jar over the 90-minute time span. The temperature inside the milling jar was recorded using thermography immediately after opening the jar at each time point (0, 2.5, 5, 7.5, 10, and 90 minutes). The experiment was performed in triplicate, and the results showed a rapid increase in internal temperature, reaching 69 ± 4°C within just 10 minutes, followed by stabilization at a plateau (Fig. S3). This indicates that the reaction proceeds under stable temperature conditions for the majority of the reaction time.

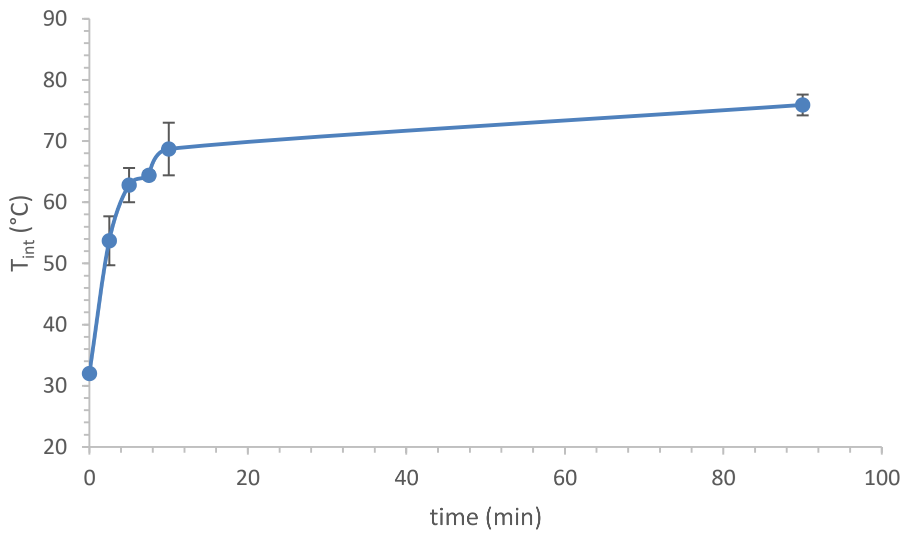

Fig. S3 : Evolution of the internal temperature of the jar over time

SI-§3. General Procedure for Solid-State C–N Coupling Using a Ball Mill.

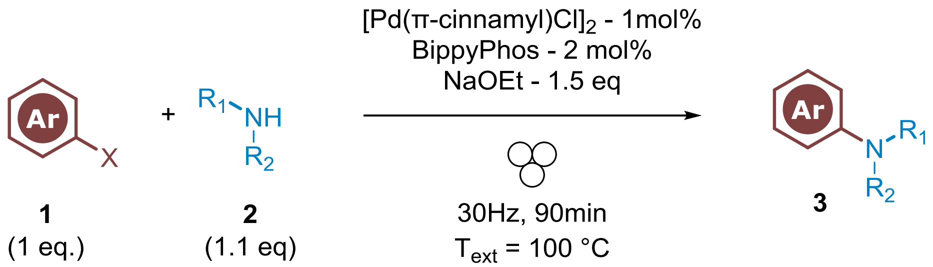

Aryl halide **1** (1 eq, 0.22 mmol), amine **2** (1.1 eq, 0.24 mmol), $[Pd(cinnamyl)Cl]_2$ (1 mol%), BippyPhos (2 mol%) and NaOEt (1.5 eq, 0.33 mmol) were placed in a ball milling vessel (stainless steel, 5 ml) loaded with three grinding balls [stainless, diameter: 10 mm] in air. The grinding vessel was closed in air and placed into a mixer ball mill (Retsch MM400, 30Hz). Heat guns positioned at a predetermined distance were set to 100 °C to provide external heating. The reaction mixture was milled for 90 minutes, after which it was extracted with EtOAc (2 × 5 mL). The extract was then passed through a Celite plug, eluting with EtOAc (10 mL). The volatiles were removed under reduced pressure, and the resulting crude product was further purified through column chromatography using the adequate eluent system to give the corresponding products **3a-3ac.**

SI-§4. Procedure for Solid-State C–N Coupling on a Gram Scale.

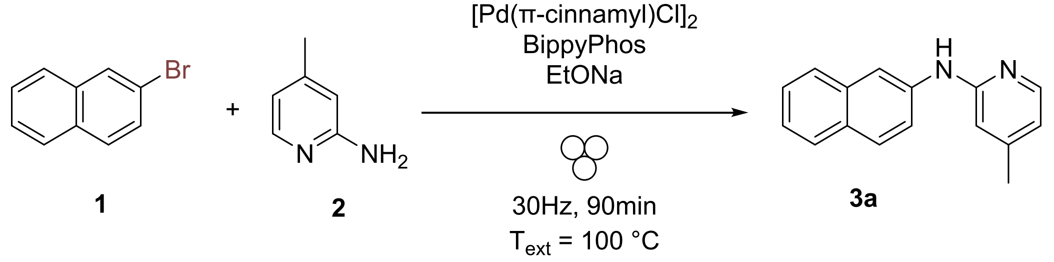

2-bromonaphthalene (1 eq., 1035 mg, 5 mmol), 2-amino-4-picoline (1.1 eq., 595 mg, 5.5 mmol), BippyPhos (0.02 eq., 50.7 mg, 0.1 mmol), $[Pd(cinnamyl)Cl]_2$ (0.01 eq., 25.9 mg, 0.05 mmol) and EtONa (1.5 eq., 510.4 mg, 588 µL, 7.5 mmol), were placed in a ball millling vessel (stainless steel, 10 ml) loaded with three grinding balls [stainless, diameter: 10 mm] in air. The grinding vessels was closed in air and placed into a mixer ball mill (Retsch MM400, 30Hz) and the heat guns placed in a pre-determined distance were set to 100 °C . After 90 minutes, the mixture was extracted with EtOAc (10 mL), and filtered. The volatiles were removed under reduced pressure, and the resulting crude mixture was sonicated in EtOH (7 mL) to afford **3a** as a solid in 83% yield (967 mg, 4.1 mmol). HPLC analysis reported a purity > 98% at 254 nm. ^{1}H NMR (400 MHz, $CDCl_3$) δ 7.98 (d, J = 5.2 Hz, 1H), 7.68 – 7.55 (m, 4H), 7.40 (s, 1H), 7.32 – 7.23 (m, 2H), 7.20 (ddd, J = 8.1, 6.8, 1.3 Hz, 1H), 6.63 (s, 1H), 6.43 (dd, J = 5.2, 1.5 Hz, 1H), 2.08 (s, 3H). ^{13}C NMR (101 MHz, $CDCl_3$) δ 156.4, 149.0, 148.1, 138.5, 134.5, 130.0, 129.1, 127.7, 127.1, 126.4, 124.3, 121.6, 116.7, 115.7, 108.9, 21.3; HRMS(ESI) m/z: $[M+H]^+$ calculated for $C_{16}H_{14}N_2$: 234.1157, found 234.1167.

Calculation of Metrics

Atom economy : $AE = \frac{\text{FW(product)}}{\sum(\text{FW(reactants)})} * 100 = 61\%$

Calculation of the E-factor (without work-up & purification)

E-factor $= \frac{\sum m(\text{Wastes})}{\text{m(Product)}} = \frac{\sum m(\text{reactants}) - m(Product)}{\text{m(Product)}} = 0.77$

Calculation of the complete E-factor (including work-up & purification)

$$\text{cE-factor} = \frac{\sum m(\text{Wastes})}{\text{m(Product)}} = \frac{\sum m(\text{reactants})+\sum m(\text{solvents})-m(Product)}{\text{m(Product)}} = 16$$

Calculation of the Process Mass Efficiency (PMI)

PMI = cE-Factor + 1 = 17

SI-§5. Solid-state NMR analysis

MAS Solid-state NMR experiments were carried out on a Bruker Avance III WB 500 MHz (11.7 T) spectrometer (Bruker Biospin, Rheinstetten, Germany) operating at a frequency of 500 MHz for ^{1}H, 202.4 MHz for ^{31}P using a 3.2 mm triple resonance (^{1}H/^{31}P/^{13}C) MAS probe (BrukerTM). The MAS frequency was set to 22.5 kHz and the effective sample temperature 303 K.

^{31}P 1D ssNMR: ^{31}P{1H} CP/MAS spectra were acquired according to the APHH scheme (adiabatic passage through the Hartmann-Hahn conditions).[1] The ^{1}H RF field was swept from 86 to 106 kHz through the Hartmann-Hahn n = 1 condition (96 kHz for ^{1}H, 73.5 kHz for ^{31}P) with a CP contact time of 3 ms. Proton decoupling during Acquisition was achieved using the SPINAL-64 scheme with an RF field of 85 kHz, and a recycling delay of 3s. The spectral width was set to 200 kHz, and 32,768 transients were acquired over 16,384-time domain data points (spectral resolution = 6.103 Hz/pt). A 60 Hz Lorentzian filer was applied prior to Fourier transform.

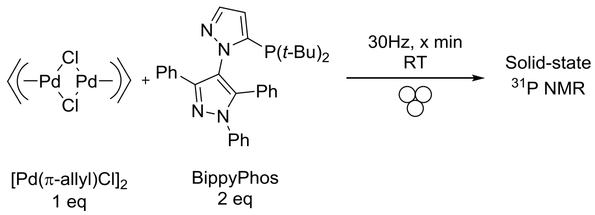

Conditions: [Pd(π-allyl)Cl]2 (0.041 mmol), BippyPhos (0.082 mmol) in a stainless-steel ball-milling jar (5 mL) with 1 stainless-steel bead (10 mm) for 30 min at 30Hz at rt. The resulting crude mixture was transferred in air to solid-state NMR rotor (3.2 mm) and studied by ^{31}P NMR (top) in comparison with solid BippyPhos alone (bottom) which was separately grinded at RT for

5 minutes at RT. NMR conditions: ^{31}P CP (Cross-polarization), Contact time 3ms, field 500 MHz, MAS frequency 22.5 kHz, Temperature 303 K.

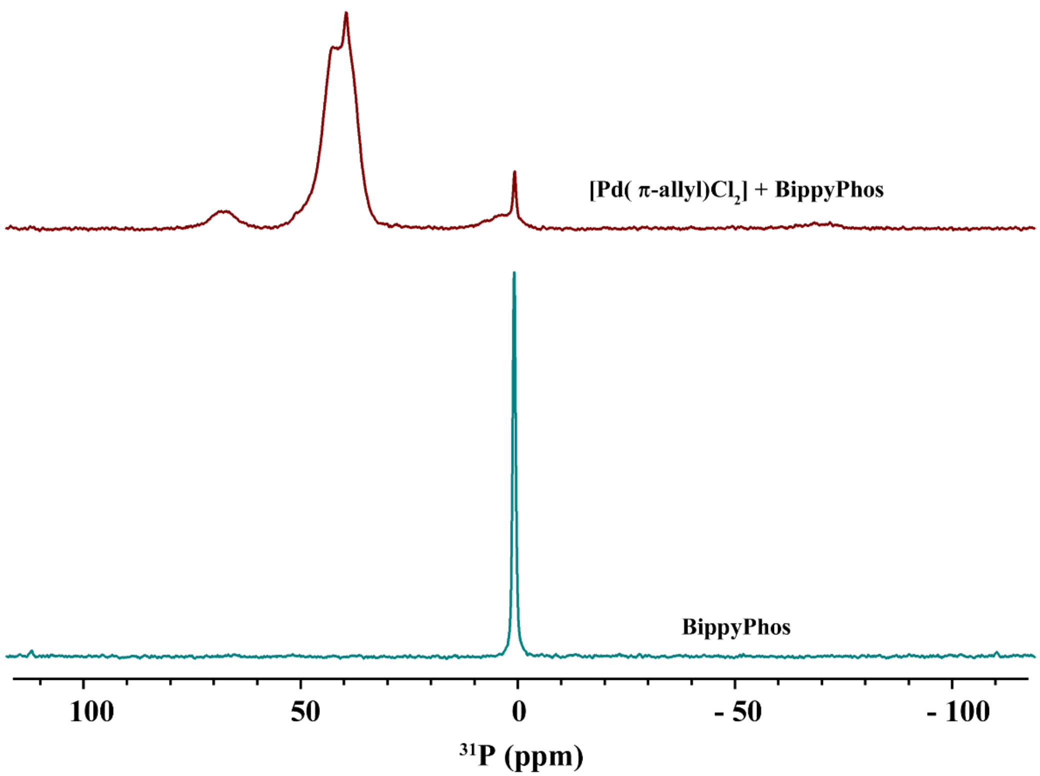

SI-§6. Characterization of the compounds 3a-ac

All compounds (3a–z) synthesized in this study are known compounds. 1H and 13C NMR were in good agreement with the reported values in the literature.

a. *4-methyl-N-(m-tolyl)pyridin-2-amine (**3b**)*

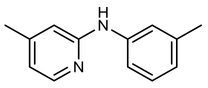

The product **3b** was synthesized following the general procedure. The crude product was purified via column chromatography on silica gel (DCM/EtOAc 10% to 25%) yielding the final product as a light-yellow oil in quantitative yield. ^{1}H NMR (400 MHz, $CDCl_3$) δ 8.07 (d, *J* = 5.2 Hz, 1H), 7.22 (t, *J* = 7.6 Hz, 1H), 7.16 – 7.10 (m, 3H), 6.87 (d, *J* = 7.6 Hz, 1H), 6.75 – 6.70 (m, 1H), 6.56 (dd, *J* = 5.1, 1.4 Hz, 1H), 2.36 (s, 3H), 2.25 (s, 3H). ^{13}C NMR (101 MHz, $CDCl_3$) δ 156.5, 148.8, 148.0, 140.7, 139.2, 129.1, 123.6, 121.3, 117.7, 116.3, 108.4, 21.5, 21.3, 21.0; HRMS(ESI) m/z: $[M+H]^+$ calculated for $C_{13}H_{15}N_2$: 199.1235, found 199.1238.

b. *N-(3-methoxyphenyl)-4-methylpyridin-2-amine (**3c**)*

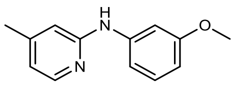

The product **3c** was synthesized following the general procedure. The crude product was purified via column chromatography on silica gel (DCM/EtOAc 10% to 25%) yielding the final compound as a colorless solid in quantitative yield. ^{1}H NMR (400 MHz, $CDCl_3$) δ 8.10 (d, *J* = 5.2 Hz, 1H), 7.26 (t, *J* = 8.1 Hz, 1H), 6.98 (t, *J* = 2.3 Hz, 1H), 6.91 (ddd, *J* = 8.0, 2.0, 0.9 Hz, 1H), 6.77 (s, 2H), 6.67 – 6.58 (m, 2H), 3.84 (s, 3H), 2.29 (s, 3H); ^{13}C NMR (101 MHz, $CDCl_3$) δ 160.5, 156.2, 148.9, 147.9, 142.1, 129.9, 116.6, 112.8, 108.9, 107.8, 106.2, 55.3, 21.3. ^{1}H and ^{13}C were consistent with the reported literature data.[2]

c. N-(4-methoxyphenyl)-4-methylpyridin-2-amine ***(3d)***

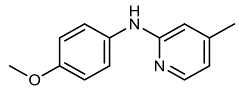

The product **3d** was synthesized following the general procedure. The crude product was purified via column chromatography on silica gel (DCM/EtOAc 10% to 25%) yielding the final compound as a light-yellow powder in 97% yield. ^{1}H NMR (400 MHz, $CDCl_3$) δ 8.04 (d, J = 5.3 Hz, 1H), 7.27 (d, J = 8.9 Hz, 2H), 6.90 – 6.97 (m, 3H), 6.57 – 6.52 (m, 2H), 3.85 (s, 3H), 2.25 (s, 3H). ^{13}C NMR (101 MHz, $CDCl_3$) δ 157.6, 156.2, 148.9, 147.8, 133.4, 124.2, 115.7, 114.6, 107.4, 55.5, 21.3. ^{1}H and ^{13}C were consistent with the reported literature data.[2]

d. N-phenylnaphthalen-2-amine ***(3e)***

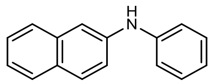

The product **3e** was synthesized following the general procedure. The crude product was purified via column chromatography on silica gel (Heptane/DCM 25%) to give the final product as a beige powder in 83% yield. ^{1}H NMR (400 MHz, $CDCl_3$) δ 7.84 (dd, J = 8.6, 2.5 Hz, 2H), 7.74 (d, J = 8.2 Hz, 1H), 7.54 – 7.46 (m, 2H), 7.45 – 7.36 (m, 3H), 7.30 (dd, J = 8.8, 2.3 Hz, 1H), 7.29 – 7.21 (m, 2H), 7.08 (tt, J = 7.3, 1.1 Hz, 1H), 5.92 (s, 1H). ^{13}C NMR (101 MHz, $CDCl_3$) δ 143.0, 140.9, 134.7, 129.5, 129.3, 129.2, 127.7, 126.6, 126.5, 123.6, 121.5, 120.1, 118.3, 111.7. ^{1}H and ^{13}C were consistent with the reported literature data.[3]

e. N-mesitylnaphthalen-2-amine ***(3f)***

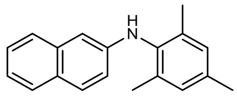

The product **3f** was synthesized following the general procedure. The crude product was purified via column chromatography on silica gel (Heptane/EtOAc 5 to 25%) to give the final product as a brown oil in 83% yield. ^{1}H NMR (400 MHz, $CDCl_3$) δ 7.67 (dd, J = 7.8, 7.8 Hz, 2H), 7.49 (d, J = 8.2 Hz, 1H), 7.31 (dd, J = 7.6, 7.6 Hz, 1H), 7.18 (dd, J = 7.5, 7.5 Hz, 1H), 6.99 (s, 2H), 6.93 (d, J = 8.7 Hz, 1H), 6.55 (s, 1H), 5.23 (s, 1H), 2.34 (s, 3H), 2.20 (s, 6H). ^{13}C NMR (101

MHz, $CDCl_3$) δ 144.4, 136.1, 135.7, 135.4, 135.2, 129.4, 129.2, 127.9, 127.7, 126.3, 126.0, 122.2, 117.5, 105.9, 21.0, 18.3. ^{1}H and ^{13}C were consistent with the reported literature data.[4]

*f. N-(3,5-dimethoxyphenyl)naphthalen-2-amine (**3g**)*

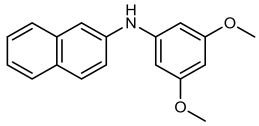

The product **3g** was synthesized following the general procedure. The crude product was purified via column chromatography on silica gel (Heptane/DCM 50 to 75%) to give the final product as a solid in 83% yield. ^{1}H NMR (400 MHz, $CDCl_3$) δ 7.77 (dd, *J* = 8.6, 3.2 Hz, 2H), 7.68 (d, *J* = 8.1 Hz, 1H), 7.49 (s, 1H), 7.44 (t, *J* = 7.0 Hz, 1H), 7.35 (t, *J* = 7.4 Hz, 1H), 7.27 (dd, *J* = 8.9, 2.2 Hz, 1H), 6.35 (d, *J* = 2.2 Hz, 2H), 6.16 (t, *J* = 2.1 Hz, 1H), 5.91 (s, 1H), 3.79 (s, 6H). ^{13}C NMR (101 MHz, $CDCl_3$) δ 161.7, 145.2, 140.4, 134.6, 129.4, 129.2, 127.7, 126.7, 126.5, 123.8, 120.7, 113.1, 96.3, 93.4, 55.4; HRMS(ESI) m/z: $[M+H]^+$ calculated for $C_{18}H_{18}NO_2$: 280.1338, found 280.1345.

*g. 4-methyl-N-(pyridin-3-yl)pyridin-2-amine (**3h**)*

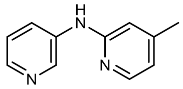

The product **3h** was synthesized following the general procedure. The crude product was purified via column chromatography on silica gel (Heptane/DCM 50 to 75%) to give the final product as a white solid in quantitative yield. ^{1}H NMR (400 MHz, DMSO) δ 9.14 (s, 1H), 8.81 (s, 1H), 8.23 (dq, *J* = 8.3, 2.2 Hz, 1H), 8.09 (s, 3H), 8.05 (d, *J* = 5.2 Hz, 1H), 7.27 (dd, *J* = 8.4, 4.6 Hz, 1H), 6.68 (s, 4H), 6.65 (d, *J* = 5.0 Hz, 1H), 2.25 (s, 3H). ^{13}C NMR (101 MHz, MeOD) δ 157.1, 150.4, 147.9, 141.7, 140.6, 127.0, 125.1, 118.1, 112.4, 21.1. ^{1}H and ^{13}C were consistent with the reported literature data.[5]

*h. N-(6-methoxynaphthalen-2-yl)-4-methylpyridin-2-amine (**3i**)*

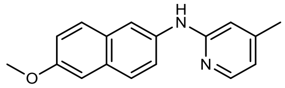

The product **3i** was synthesized following the general procedure. The crude product was purified via column chromatography on silica gel (DCM/EtOAc 0 to 10%) to give the final product as a light beige powder in 71% yield. ^{1}H NMR (400 MHz, $CDCl_3$) δ 8.25 (d, *J* = 5.2 Hz, 1H), 7.88 (d, *J* = 2.2 Hz, 1H), 7.85 (d, *J* = 8.7 Hz, 1H), 7.80 (d, *J* = 8.9 Hz, 1H), 7.54 (dd, *J* = 8.7, 2.3 Hz, 1H), 7.32 – 7.23 (m, 2H), 6.86 (s, 1H), 6.72 (d, *J* = 5.1 Hz, 1H), 4.05 (s, 3H), 2.39 (s, 3H). ^{13}C NMR (101 MHz, $CDCl_3$) δ 156.9, 149.0, 148.1, 136.4, 131.2, 129.8, 128.6, 127.9, 122.7, 119.2, 117.2, 116.4, 108.4, 106.0, 55.4, 21.4; HRMS(ESI) m/z: $[M+H]^+$ calculated for $C_{17}H_{17}N_2O$: 265.1341, found 265.1348.

*i. 4-methyl-N-(3-(trifluoromethyl)phenyl)pyridin-2-amine (**3j**)*

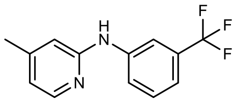

The product **3j** was synthesized following the general procedure. The crude product was purified via column chromatography on silica gel (DCM/EtOAc 0 to 10%) to give the final product as a light-yellow powder in 86% yield. ^{1}H NMR (400 MHz, $CDCl_3$) δ 7.97 (d, *J* = 5.2 Hz, 1H), 7.50 (t, *J* = 2.1 Hz, 1H), 7.41 (dd, *J* = 8.3, 2.2 Hz, 1H), 7.26 (t, *J* = 7.9 Hz, 1H), 7.14 (s, 1H), 7.10 (d, *J* = 7.8 Hz, 1H), 6.54 (s, 1H), 6.50 (d, *J* = 5.1 Hz, 1H), 2.14 (s, 3H). ^{19}F NMR (376 MHz, $CDCl_3$) δ -62.7. ^{13}C NMR (101 MHz, $CDCl_3$) δ 155.47, 149.20, 147.91, 141.53, 131.55 (q, *J* = 32.1 Hz), 129.65, 124.10 (d, *J* = 272.4 Hz), 122.45, 122.44, 118.53, 118.49, 117.37, 116.03, 115.99, 109.48, 21.21; HRMS(ESI) m/z: $[M+H]^+$ calculated for $C_{13}H_{12}F_3N_2$: 253.0953, found 253.0960.

*j. 3-(naphthalen-2-ylamino)benzonitrile (**3k**)*

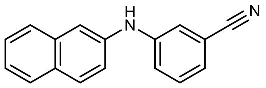

The product **3k** was synthesized following the general procedure. The crude product was purified via column chromatography on silica gel (Heptane/DCM 50 to 100%) to give the final product as a beige solid in 64% yield. ^{1}H NMR (400 MHz, $CDCl_3$) δ 7.78 – 7.72 (m, 2H), 7.66 (dd, *J* = 8.2, 1.1 Hz, 1H), 7.45 – 7.39 (m, 2H), 7.34 (ddd, *J* = 8.1, 6.9, 1.3 Hz, 1H), 7.31 – 7.25 (m, 2H), 7.22 (ddd, *J* = 8.1, 2.5, 1.3 Hz, 1H), 7.21 – 7.16 (m, 1H), 7.12 (dt, *J* = 7.2, 1.4 Hz, 1H), 6.00 (s, 1H). ^{13}C NMR (101 MHz, $CDCl_3$) δ 144.5, 138.8, 134.4, 130.3, 130.1, 129.6, 127.8, 126.8, 126.8,

124.5, 123.9, 121.0, 120.7, 119.3, 114.6, 113.2; HRMS(ESI) m/z: $[M+H]^+$ calculated for $C_{17}H_{13}N_2$: 245.1079, found 245.1077.

*N-(4-nitrophenyl)naphthalen-2-amine (**3l**)*

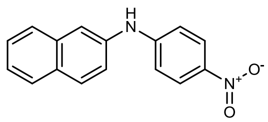

The product **3l** was synthesized following the general procedure. The crude product was purified via column chromatography on silica gel (Heptane/DCM 75%) to give the final product as a bright orange powder in 97% yield. ^{1}H NMR (400 MHz, DMSO) δ 9.54 (s, 1H), 8.13 (d, J = 9.2 Hz, 2H), 7.91 (d, J = 8.8 Hz, 1H), 7.89 – 7.81 (m, 2H), 7.75 (d, J = 2.2 Hz, 1H), 7.48 (ddd, J = 8.3, 6.8, 1.3 Hz, 1H), 7.45 – 7.36 (m, 2H), 7.20 (d, J = 9.3 Hz, 2H). ^{13}C NMR (101 MHz, DMSO) δ 151.0, 138.7, 138.3, 134.3, 130.3, 129.7, 128.0, 127.4, 127.0, 126.6, 125.1, 122.0, 116.2, 114.3, 40.6, 40.4, 40.2, 40.0, 39.8, 39.6, 39.4. ^{1}H and ^{13}C were consistent with the reported literature data.[6]

*k. N-(3-(trifluoromethyl)phenyl)naphthalen-2-amine (**3m**)*

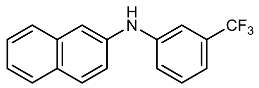

The product **3m** was synthesized following the general procedure. The crude product was purified via column chromatography on silica gel (Heptane/EtOAc 2%) to give the final product as a white powder in quantitative yield. ^{1}H NMR (400 MHz, $CDCl_3$) δ 7.81 (dd, J = 8.5, 4.1 Hz, 2H), 7.71 (d, J = 8.2 Hz, 1H), 7.52 – 7.43 (m, 2H), 7.43 – 7.33 (m, 3H), 7.32 – 7.23 (m, 2H), 7.20 (d, J = 7.7 Hz, 1H), 5.97 (s, 1H). ^{19}F NMR (376 MHz, $CDCl_3$) δ -62.8. ^{13}C NMR (101 MHz, $CDCl_3$) δ 143.9, 139.5, 134.5, 131.9 (q, J = 32.1 Hz), 129.9, 129.8, 129.5, 127.8, 126.7, 126.7, 124.2, 124.1 (d, J = 272.4 Hz), 120.5, 120.1, 117.3 (q, J = 3.9 Hz), 113.8 (q, J = 3.9 Hz), 113.7. ^{1}H and ^{13}C were consistent with the reported literature data.[4]

l. *N-(4-methylpyridin-2-yl)quinolin-2-amine (**3p**)*

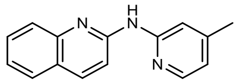

The product **3p** was synthesized following the general procedure. The crude product was purified via column chromatography on silica gel (DCM/MeOH 0 to 2.5%) to give the final product as a light-yellow powder in 38% yield. ^{1}H NMR (400 MHz, $CDCl_3$) δ 8.17 (d, *J* = 5.1 Hz, 1H), 8.13 (s, 1H), 7.97 (d, *J* = 8.9 Hz, 1H), 7.86 (d, *J* = 8.3 Hz, 1H), 7.68 (dd, *J* = 8.1, 1.5 Hz, 1H), 7.61 (ddd, *J* = 8.4, 7.0, 1.5 Hz, 1H), 7.38 – 7.30 (m, 2H), 6.77 (dd, *J* = 5.1, 1.5 Hz, 1H), 2.40 (s, 3H); ^{13}C NMR (101 MHz, $CDCl_3$) δ 154.0, 153.0, 149.3, 147.3, 137.7, 129.7, 127.5, 126.9, 124.5, 123.7, 118.6, 113.9, 113.2, 21.6. ^{1}H and ^{13}C were consistent with the reported literature data.[7]

m. *N-(naphthalen-2-yl)naphthalen-1-amine (**3q**)*

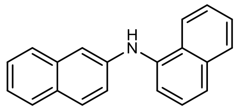

The product **3q** was synthesized following the general procedure. The crude product was purified via column chromatography on silica gel (Heptane/EtOAc 0 to 5%) to give the final product as a light-yellow oil in 38% yield. ^{1}H NMR (400 MHz, $CDCl_3$) δ 8.12 (d, *J* = 8.3 Hz, 1H), 7.97 (d, *J* = 8.0 Hz, 1H), 7.85 – 7.78 (m, 2H), 7.74 – 7.63 (m, 2H), 7.63 – 7.41 (m, 4H), 7.37 (t, *J* = 7.5 Hz, 1H), 7.34 – 7.25 (m, 2H), 6.12 (s, 1H). ^{13}C NMR (101 MHz, $CDCl_3$) δ 142.7, 138.7, 134.8, 134.8, 129.2, 129.1, 128.7, 128.1, 127.8, 126.5, 126.5, 126.3, 126.1, 125.9, 123.5, 123.4, 122.0, 119.8, 116.7, 111.4. ^{1}H and ^{13}C were consistent with the reported literature data.[8]

n. *N-(naphthalen-2-yl)pyrimidin-2-amine (**3r**)*

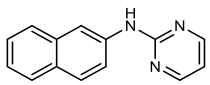

The product **3r** was synthesized following the general procedure. The crude product was purified via column chromatography on silica gel (Heptane/DCM 50% to 100%) to give the final product

as a light beige powder in 82% yield. ^{1}H NMR (400 MHz, $CDCl_3$) δ 8.48 (d, J = 4.8 Hz, 2H), 8.30 (d, J = 2.2 Hz, 1H), 7.85 – 7.75 (m, 3H), 7.57 (dd, J = 8.8, 2.3 Hz, 1H), 7.50 – 7.41 (m, 2H), 7.37 (ddd, J = 8.1, 6.9, 1.3 Hz, 1H), 6.77 (t, J = 4.8 Hz, 1H). ^{13}C NMR (101 MHz, $CDCl_3$) δ 160.3, 158.1, 137.0, 134.2, 130.0, 128.6, 127.6, 127.4, 126.3, 124.3, 120.6, 115.3, 112.7. ^{1}H and ^{13}C were consistent with the reported literature data.[9]

*o. N-methyl-N-phenylnaphthalen-2-amine (**3s**)*

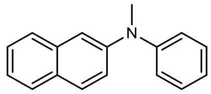

The product **3s** was synthesized following the general procedure. The crude product was purified via column chromatography on silica gel (Heptane/EtOAc 2%) to give the final product as a beige solid in 74% yield. ^{1}H NMR (400 MHz, $CDCl_3$) δ 7.88 (d, J = 8.2 Hz, 1H), 7.83 (t, J = 8.5 Hz, 2H), 7.56 (ddd, J = 8.2, 6.9, 1.3 Hz, 1H), 7.49 – 7.41 (m, 4H), 7.36 (dd, J = 8.9, 2.4 Hz, 1H), 7.25 (dd, J = 8.7, 1.2 Hz, 2H), 7.16 (tt, J = 7.3, 1.2 Hz, 1H), 3.56 (s, 3H). ^{13}C NMR (101 MHz, $CDCl_3$) δ 149.2, 146.8, 134.8, 129.4, 129.3, 128.7, 127.7, 126.9, 126.4, 123.8, 122.1, 121.9, 121.5, 114.7, 40.7. ^{1}H and ^{13}C were consistent with the reported literature data.[10]

*p. N-benzylnaphthalen-2-amine (**3t**)*

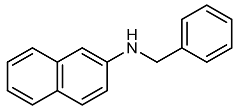

The product **3t** was synthesized following the general procedure. The crude product was purified via column chromatography on silica gel (Heptane/DCM 25%) to give the final product as a white solid in 94% yield. ^{1}H NMR (400 MHz, $CDCl_3$) δ 7.70 (dd, J = 8.2, 1.2 Hz, 1H), 7.66 (d, J = 8.8 Hz, 1H), 7.62 (dd, J = 8.3, 1.1 Hz, 1H), 7.48 – 7.42 (m, 2H), 7.43 – 7.34 (m, 3H), 7.35 – 7.29 (m, 1H), 7.23 (ddd, J = 8.1, 6.8, 1.2 Hz, 1H), 6.94 (dd, J = 8.7, 2.4 Hz, 1H), 6.87 (d, J = 2.4 Hz, 1H), 4.45 (s, 2H), 4.21 (s, 1H). ^{13}C NMR (101 MHz, $CDCl_3$) δ 145.8, 139.3, 135.3, 129.0, 128.8, 127.7, 127.7, 127.4, 126.4, 126.1, 122.1, 117.9, 104.7, 48.4. ^{1}H and ^{13}C were consistent with the reported literature data.[11]

q. *N-phenethylnaphthalen-2-amine* (**3u**)

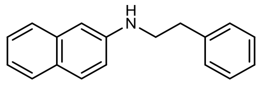

The product **3u** was synthesized following the general procedure. The crude product was purified via column chromatography on silica gel (Heptane/DCM 50%) to give the final product as a light brown oil in 83% yield. ^{1}H NMR (400 MHz, $CDCl_3$) δ 7.68 (d, *J* = 8.1 Hz, 1H), 7.63 (dd, *J* = 8.5, 4.8 Hz, 2H), 7.41 – 7.30 (m, 3H), 7.29 – 7.18 (m, 4H), 6.88 – 6.79 (m, 2H), 3.82 (s, 1H), 3.49 (t, *J* = 7.0 Hz, 2H), 2.97 (t, *J* = 7.0 Hz, 2H). ^{13}C NMR (101 MHz, $CDCl_3$) δ 145.5, 139.3, 135.3, 129.0, 128.9, 128.7, 127.7, 126.6, 126.4, 126.0, 122.2, 118.2, 105.0, 77.4, 77.1, 76.8, 45.2, 35.3. ^{1}H and ^{13}C were consistent with the reported literature data.[12]

r. *N-(naphthalen-2-yl)adamantan-1-amine (3v)*

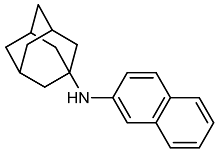

The product **3v** was synthesized following the general procedure. The crude product was purified via column chromatography on silica gel (Heptane/DCM 10%) to give the final product as a pale yellow solid in 46% yield. ^{1}H NMR (400 MHz, $CDCl_3$) δ 7.69 (d, *J* = 8.1 Hz, 1H), 7.63 (dd, *J* = 8.5, 4.3 Hz, 2H), 7.37 (ddd, *J* = 8.2, 6.8, 1.3 Hz, 1H), 7.23 (ddd, *J* = 8.1, 6.8, 1.2 Hz, 1H), 7.13 (d, *J* = 2.3 Hz, 1H), 6.97 (dd, *J* = 8.8, 2.4 Hz, 1H), 2.15 (s, 2H), 1.99 (d, *J* = 2.9 Hz, 7H), 1.72 (d, *J* = 3.3 Hz, 6H). ^{13}C NMR (101 MHz, $CDCl_3$) δ 143.6, 134.7, 128.4, 128.1, 127.5, 126.2, 126.1, 122.5, 122.0, 111.9, 52.5, 43.3, 36.5, 29.8. ^{1}H and ^{13}C were consistent with the reported literature data.[13]

s. *1-(naphthalen-2-yl)piperidine (3w)*

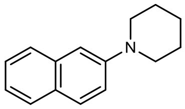

The product **3w** was synthesized following the general procedure. The crude product was purified via column chromatography on silica gel (Heptane/DCM 50%) to give the final product as an off-white powder in 81% yield. ^{1}H NMR (400 MHz, $CDCl_3$) δ 7.68 (dd, *J* = 9.1, 9.1 Hz,

3H), 7.37 (ddd, J = 8.2, 6.8, 1.3 Hz, 1H), 7.31 – 7.22 (m, 2H), 7.12 (d, J = 2.5 Hz, 1H), 3.28 – 3.20 (m, 4H), 1.79 – 1.71 (m, 4H), 1.66 – 1.55 (m, 2H). ^{13}C NMR (101 MHz, $CDCl_3$) δ 150.1, 134.8, 128.5, 128.4, 127.4, 126.7, 126.1, 123.1, 120.2, 110.4, 51.1, 25.9, 24.4. ^{1}H and ^{13}C were consistent with the reported literature data.[14]

*t. 1-benzyl-4-(naphthalen-2-yl)piperazine (**3x**)*

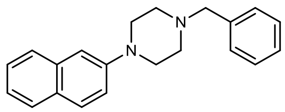

The product **3x** was synthesized following the general procedure. The crude product was purified via column chromatography on silica gel (DCM/EtOAc 2.5%) to give the final product as a light-beige powder in 63% yield. ^{1}H NMR (400 MHz, $CDCl_3$) δ 7.68 – 7.52 (m, 3H), 7.33 – 7.20 (m, 5H), 7.20 – 7.12 (m, 3H), 7.00 (d, J = 2.5 Hz, 1H), 3.49 (s, 2H), 3.23 – 3.16 (m, 4H), 2.60 – 2.53 (m, 4H). ^{13}C NMR (101 MHz, $CDCl_3$) δ 149.3, 138.0, 134.7, 129.3, 128.7, 128.5, 128.4, 127.5, 127.2, 126.8, 126.3, 123.4, 119.4, 110.2, 63.1, 53.1, 49.6.^{1}H and ^{13}C were consistent with the reported literature data.[15]

*u. tert-butyl 4-(naphthalen-2-yl)piperazine-1-carboxylate (**3y**)*

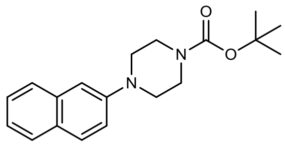

The product **3y** was synthesized following the general procedure. The crude product was purified via column chromatography on silica gel (Hept/EtOAc 5%) to give the final product as a light-beige powder in 64% yield. ^{1}H NMR (400 MHz, $CDCl_3$) δ 7.91 – 7.80 (m, 3H), 7.55 (ddd, J = 8.2, 6.8, 1.3 Hz, 1H), 7.45 (ddd, J = 8.1, 6.9, 1.3 Hz, 1H), 7.39 (dd, J = 9.0, 2.5 Hz, 1H), 7.26 (d, J = 2.4 Hz, 1H), 3.85 – 3.68 (m, 4H), 3.42 – 3.31 (m, 4H), 1.65 (s, 9H). ^{13}C NMR (101 MHz, $CDCl_3$) δ 154.8, 149.1, 134.5, 128.9, 128.8, 127.5, 126.8, 126.4, 123.7, 119.8, 110.9, 79.9, 49.8, 43.7, 28.5. ^{1}H and ^{13}C were consistent with the reported literature data.[16]

*v. tert-butyl naphthalen-2-ylcarbamate (**3z**)*

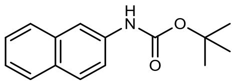

The product **3z** was synthesized following the general procedure. The crude product was purified via column chromatography on silica gel (Heptane/EtOAc 2.5 to 5%) to give the final product as a pale yellow solid in 34% yield. ^{1}H NMR (400 MHz, $CDCl_3$) δ 8.00 (s, 1H), 7.76 (dt, J = 7.9, 1.1 Hz, 3H), 7.44 (ddd, J = 8.0, 6.7, 1.3 Hz, 1H), 7.41 – 7.31 (m, 2H), 7.26 (s, 0H), 6.67 (s, 1H), 1.56 (s, 9H). ^{13}C NMR (101 MHz, $CDCl_3$) δ 152.9, 135.8, 134.1, 130.0, 128.7, 127.5, 127.4, 126.4, 124.5, 119.2, 114.6, 80.7, 28.4. ^{1}H and ^{13}C were consistent with the reported literature data.[17]

*w. 1,1-dimethyl-3-(naphthalen-2-yl)urea (**3aa**)*

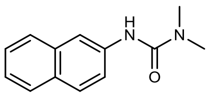

The product **3aa** was synthesized following the general procedure. The crude product was purified via column chromatography on silica gel (Heptane/EtOAc 2.5 to 5%) to give the final product as a pale yellow solid in 73% yield. ^{1}H NMR (400 MHz, DMSO-d_6) δ 8.50 (s, 1H), 8.03 (d, J = 2.3 Hz, 1H), 7.83 – 7.76 (m, 2H), 7.73 (dd, J = 8.3, 1.2 Hz, 1H), 7.64 (dt, J = 8.9, 2.0 Hz, 1H), 7.42 (ddd, J = 8.2, 6.9, 1.4 Hz, 1H), 7.34 (ddd, J = 8.1, 6.8, 1.3 Hz, 1H), 2.98 (s, 6H). ^{13}C NMR (101 MHz, DMSO-d_6) δ 156.3, 138.9, 134.0, 129.5, 128.1, 127.8, 127.4, 126.5, 124.3, 121.7, 115.3, 36.7. ^{1}H and ^{13}C were consistent with the reported literature data.[18]

*x. N-(naphthalen-2-yl)benzamide (**3ab**)*

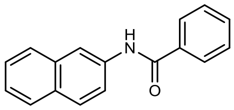

The product **3ab** was synthesized followingthe general procedure. The crude product was purified via column chromatography on silica gel (Heptane/DCM 50% to DCM 100%) to give the final product as a light beige powder in 65% yield. ^{1}H NMR (400 MHz, DMSO-d_6) δ 10.47 (s, 1H), 8.48 (d, J = 2.0 Hz, 1H), 8.07 – 7.99 (m, 2H), 7.95 – 7.81 (m, 4H), 7.68 – 7.53 (m, 3H),

7.50 (ddd, J = 8.3, 6.9, 1.4 Hz, 1H), 7.44 (ddd, J = 8.0, 6.8, 1.3 Hz, 1H). ^{13}C NMR (101 MHz, DMSO-d_6) δ 166.3, 137.3, 135.4, 133.8, 132.1, 130.5, 128.9, 128.6, 128.2, 127.9, 127.9, 126.9, 125.3, 121.4, 117.0. ^{1}H and ^{13}C were consistent with the reported literature data.[19]

*y. 4-methoxy-N-(naphthalen-2-yl)benzamide (**3ac**)*

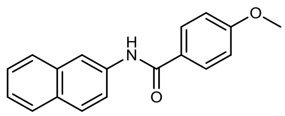

The product **3ac** was synthesized following the general procedure. The crude product was purified via column chromatography on silica gel (Heptane/DCM 50% to DCM 100%) to give the final product as a light beige solid in 90% yield. ^{1}H NMR (400 MHz, DMSO-d_6) δ 10.31 (s, 1H), 8.46 (t, J = 2.7 Hz, 1H), 8.07 – 7.99 (m, 2H), 7.93 – 7.80 (m, 4H), 7.49 (ddd, J = 8.3, 6.8, 1.3 Hz, 1H), 7.43 (ddd, J = 8.1, 6.8, 1.3 Hz, 1H), 7.14 – 7.06 (m, 2H), 3.86 (s, 3H). ^{13}C NMR (101 MHz, DMSO-d_6) δ 165.6, 162.5, 137.5, 133.9, 130.4, 130.1, 128.5, 127.9, 127.8, 127.4, 126.8, 125.1, 121.5, 116.9, 116.8, 114.1, 55.9. ^{1}H and ^{13}C were consistent with the reported literature data.[20]

SI-§7. Bibliography

(1) Hediger, S.; Meier, B. H.; Kurur, N. D.; Bodenhausen, G.; Ernst, R. R. NMR Cross Polarization by Adiabatic Passage through the Hartmann—Hahn Condition (APHH). *Chem. Phys. Lett.* **1994**, *223* (4), 283–288.

(2) Chu, J.-H.; Huang, H.-P.; Hsu, W.-T.; Chen, S.-T.; Wu, M.-J. Palladium(II)-Catalyzed Direct Ortho Arylation of 4-Methyl- *N* -Phenylpyridin-2-Amines via C–H Activation/C–C Coupling and Synthetic Applications. *Organometallics* **2014**, *33* (5), 1190–1204.

(3) Yang, J. C.; Niu, D.; Karsten, B. P.; Lima, F.; Buchwald, S. L. Use of a "Catalytic" Cosolvent, *N* , *N* -Dimethyl Octanamide, Allows the Flow Synthesis of Imatinib with No Solvent Switch. *Angew. Chem. Int. Ed.* **2016**, *55* (7), 2531–2535.

(4) Ackermann, L.; Sandmann, R.; Song, W. Palladium- and Nickel-Catalyzed Aminations of Aryl Imidazolylsulfonates and Sulfamates. *Org. Lett.* **2011**, *13* (7), 1784–1786.

(5) Salomé, C.; Wagner, P.; Bollenbach, M.; Bihel, F.; Bourguignon, J.-J.; Schmitt, M. Buchwald–Hartwig Reactions in Water Using Surfactants. *Tetrahedron* **2014**, *70* (21), 3413–3421.

(6) Zhang, Z.; Yue, S.; Jin, B.; Yang, R.; Wang, S.; Zhang, T.; Sun, L.; Lei, A.; Cai, H. Para-Selective Nitrobenzene Amination Lead by C(Sp2)-H/N-H Oxidative Cross-Coupling through Aminyl Radical. *Nat. Commun.* **2024**, *15* (1), 4186.

(7) Li, K.; Li, Y.; Zhou, D.; Fan, Y.; Guo, H.; Ma, T.; Wen, J.; Liu, D.; Zhao, L. Synthesis and Biological Evaluation of Quinoline Derivatives as Potential Anti-Prostate Cancer Agents and Pim-1 Kinase Inhibitors. *Bioorg. Med. Chem.* **2016**, *24* (8), 1889–1897.
(8) Wang, Z.-C.; Li, Y.-Y.; Zhang, S.-Q.; Hong, X.; Shi, S.-L. Unsymmetric N-Heterocyclic Carbene Ligand Enabled Nickel-Catalysed Arylation of Bulky Primary and Secondary Amines. *Chem. Sci.* **2023**, *14* (16), 4390–4396.
(9) Pratap, R.; Yorimitsu, H. Palladium-Catalyzed Amination of Aryl Sulfides and Sulfoxides with Azaarylamines of Poor Nucleophilicity. *Synthesis* **2019**, *51* (13), 2705–2712.
(10) Toupalas, G.; Morandi, B. Non-Innocent Electrophiles Unlock Exogenous Base-Free Coupling Reactions. *Nat. Catal.* **2022**, *5* (4), 324–331.
(11) Mastalir, M.; Tomsu, G.; Pittenauer, E.; Allmaier, G.; Kirchner, K. Co(II) PCP Pincer Complexes as Catalysts for the Alkylation of Aromatic Amines with Primary Alcohols. *Org. Lett.* **2016**, *18* (14), 3462–3465.
(12) Liang, W.; Xie, F.; Yang, Z.; Zeng, Z.; Xia, C.; Li, Y.; Zhu, Z.; Chen, X. Mono/Dual Amination of Phenols with Amines in Water. *Org. Lett.* **2020**, *22* (21), 8291–8295.
(13) Dhayalan, V.; Knochel, P. Synthesis of Polyfunctional Secondary Amines by the Reaction of Functionalized Organomagnesium Reagents with Tertiary Nitroalkanes. *Synthesis* **2015**, *47* (20), 3246–3256.
(14) Hatakeyama, T.; Yoshimoto, Y.; Ghorai, S. K.; Nakamura, M. Transition-Metal-Free Electrophilic Amination between Aryl Grignard Reagents and *N* -Chloroamines. *Org. Lett.* **2010**, *12* (7), 1516–1519.
(15) Ghazanfarpour-Darjani, M.; Barat-Seftejani, F.; Khalaj, M.; Mousavi-Safavi, S. M. Synthesis of *N* -alkyl- *N* ′-aryl or Alkenylpiperazines: A Copper-Catalyzed C–N Cross-Coupling in the Presence of Aryl and Alkenyl Triflates and DABCO. *Helv. Chim. Acta* **2017**, *100* (8), e1700082.
(16) Chen, S.; Wang, P.; Cheng, H.-G.; Yang, C.; Zhou, Q. Redox-Neutral *Ortho* -C–H Amination of Pinacol Arylborates *via* Palladium(II)/Norbornene Catalysis for Aniline Synthesis. *Chem. Sci.* **2019**, *10* (36), 8384–8389.
(17) Jin, W.; Trzupek, J. D.; Rayl, T. J.; Broward, M. A.; Vielhauer, G. A.; Weir, S. J.; Hwang, I.; Boger, D. L. A Unique Class of Duocarmycin and CC-1065 Analogues Subject to Reductive Activation. *J. Am. Chem. Soc.* **2007**, *129* (49), 15391–15397.
(18) Nan, J.; Ren, X.; Yan, Q.; Liu, S.; Wang, J.; Ma, Y.; Szostak, M. Hypervalent Iodine-Promoted Twofold Oxidative Coupling of Amines with Amides and Thioamides: Chemoselective Pathway to Oxazoles and Thiazoles. *Chem. Sci.* **2023**, *14* (12), 3338–3345.
(19) Chen, C.-T.; Kuo, J.-H.; Pawar, V. D.; Munot, Y. S.; Weng, S.-S.; Ku, C.-H.; Liu, C.-Y. Nucleophilic Acyl Substitutions of Anhydrides with Protic Nucleophiles Catalyzed by Amphoteric, Oxomolybdenum Species. *J. Org. Chem.* **2005**, *70* (4), 1188–1197.
(20) Naro, Y.; Thomas, M.; Stephens, M. D.; Connelly, C. M.; Deiters, A. Aryl Amide Small-Molecule Inhibitors of microRNA miR-21 Function. *Bioorg. Med. Chem. Lett.* **2015**, *25* (21), 4793–4796.

SI-§8. NMR Traces

z. Cpd **3a**

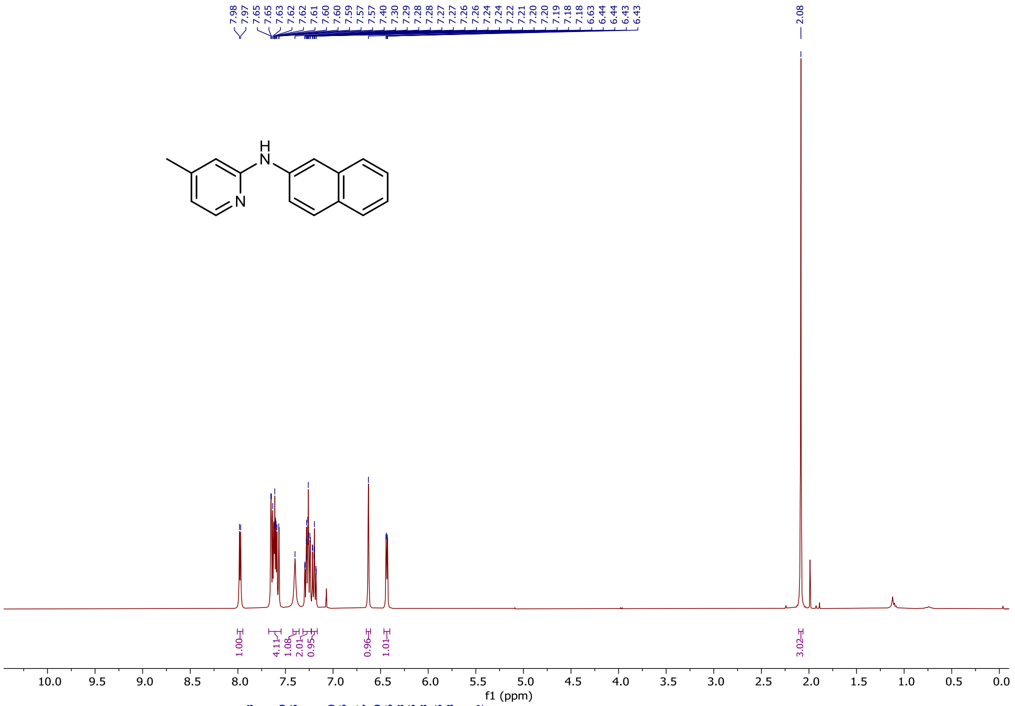

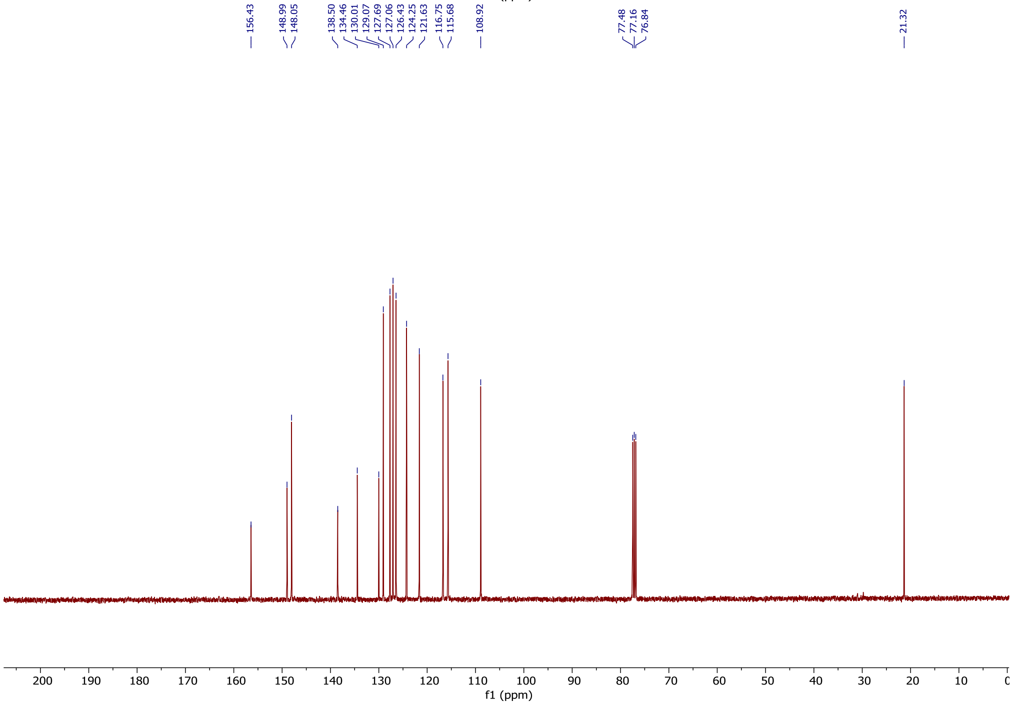

aa. *4-methyl-N-(m-tolyl)pyridin-2-amine* **3b**

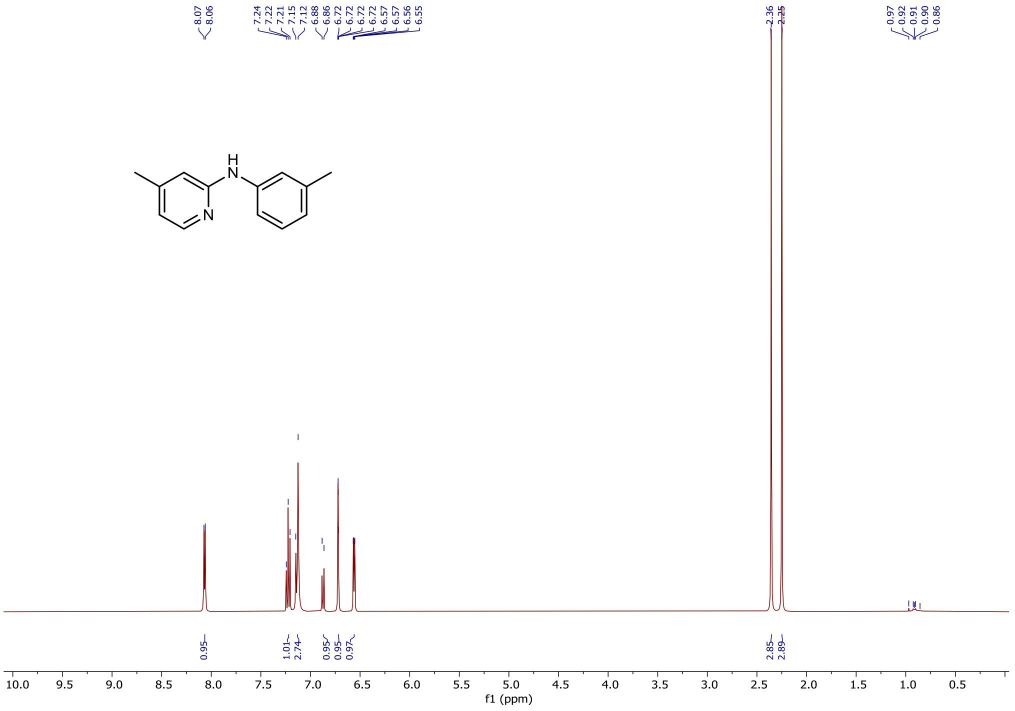

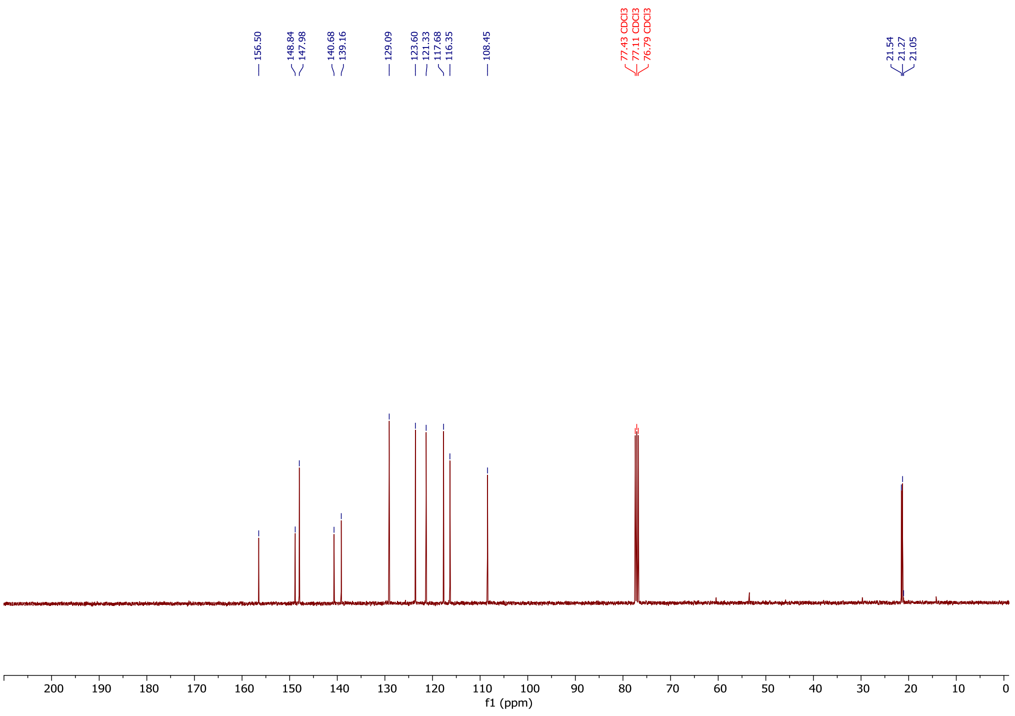

bb. *N-(3-methoxyphenyl)-4-methylpyridin-2-amine* **3c**

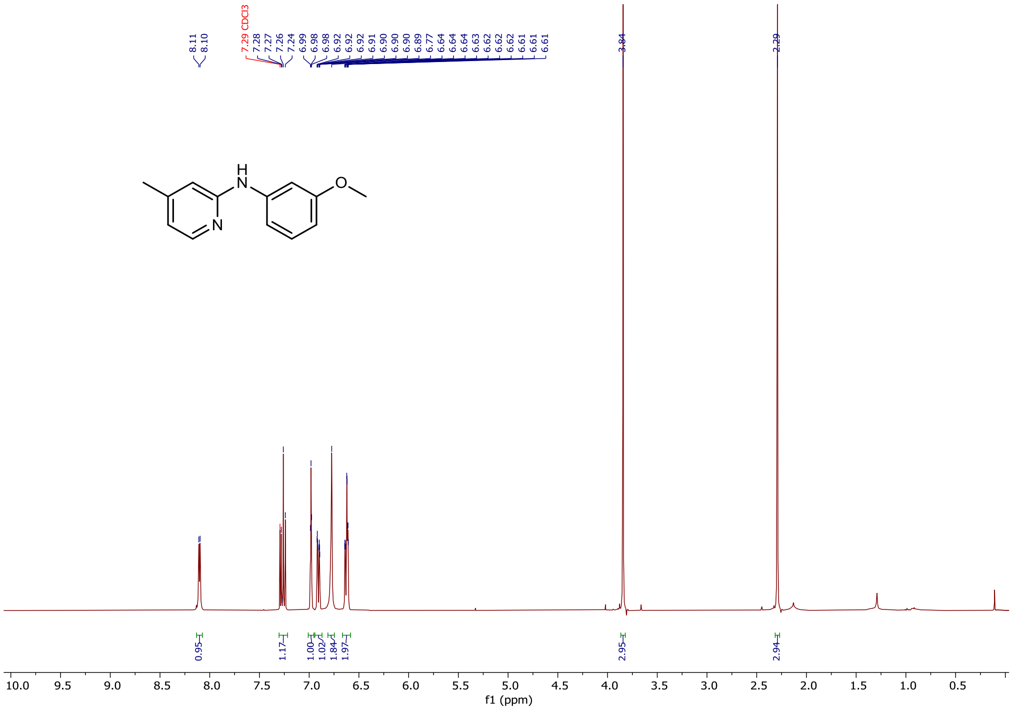

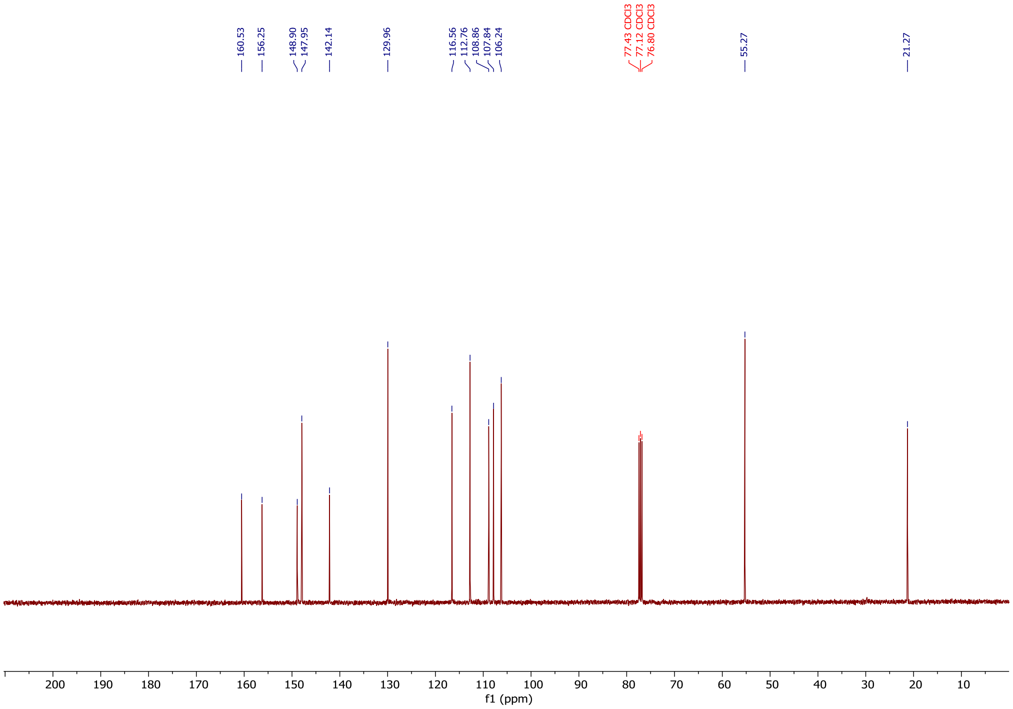

cc. *N-(4-methoxyphenyl)-4-methylpyridin-2-amine* **3d**

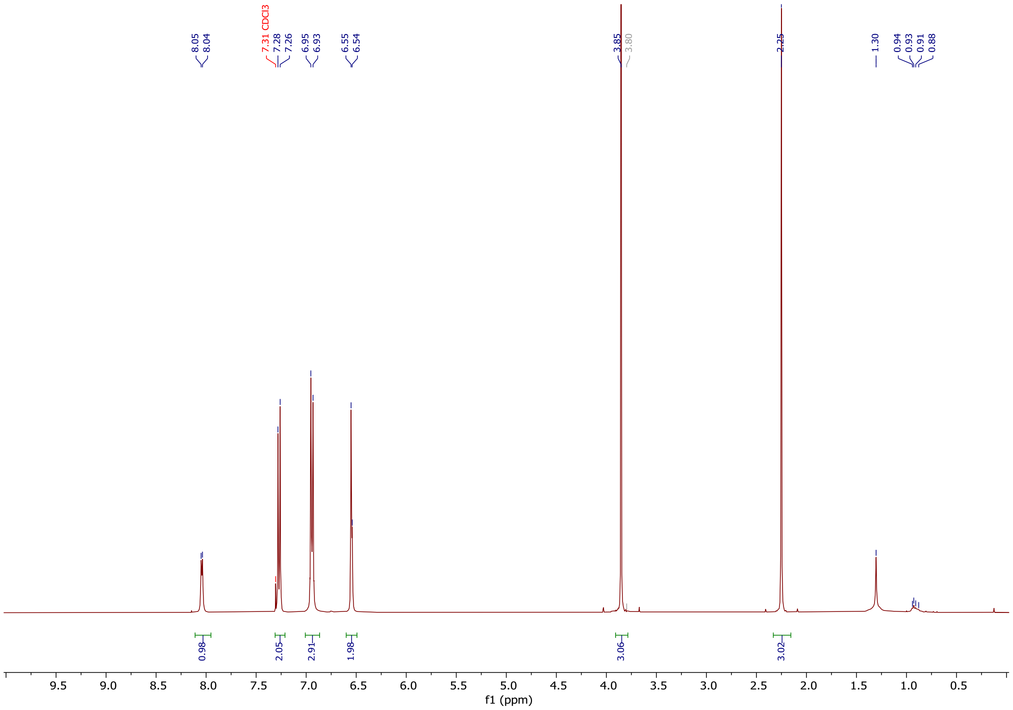

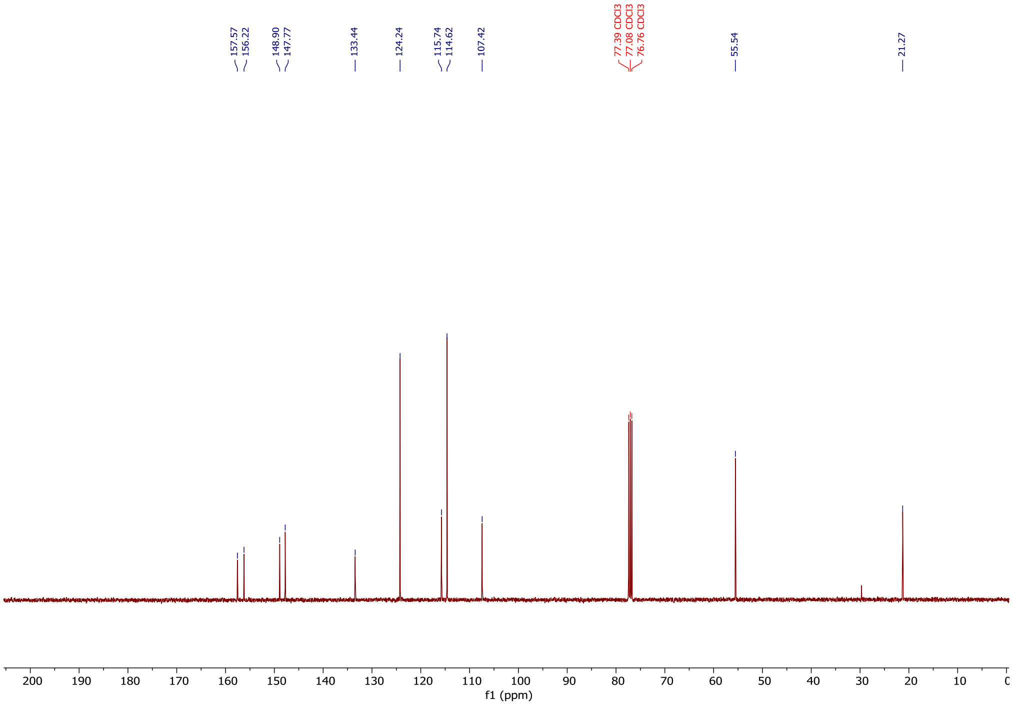

dd. *N-phenylnaphthalen-2-amine* **3e**

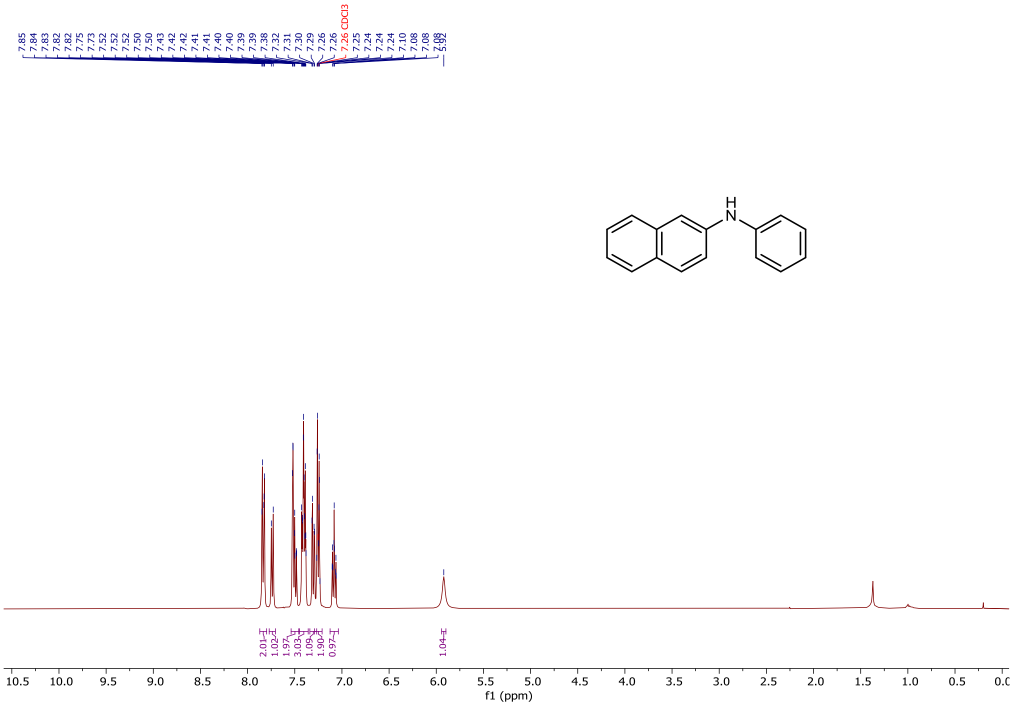

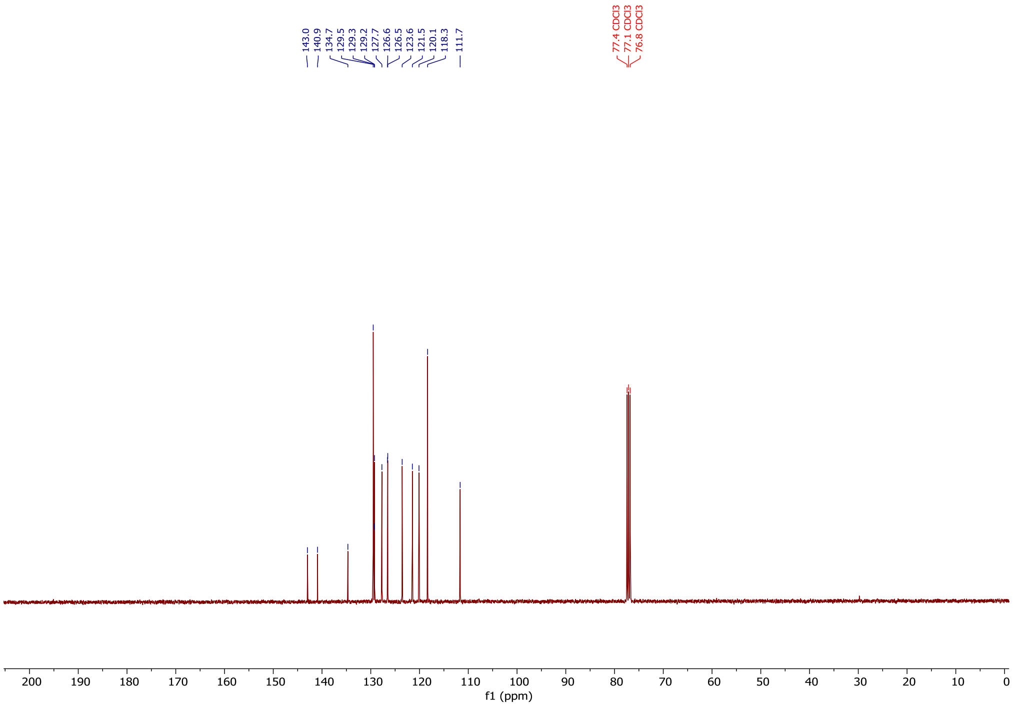

ee. *N-mesitylnaphthalen-2-amine* **3f**

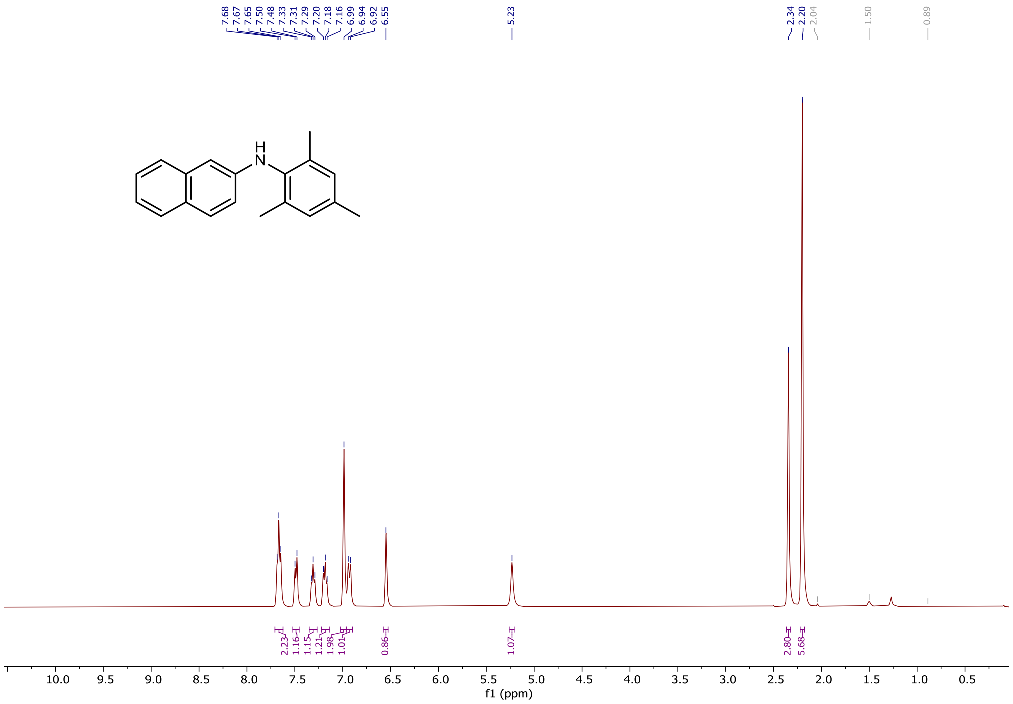

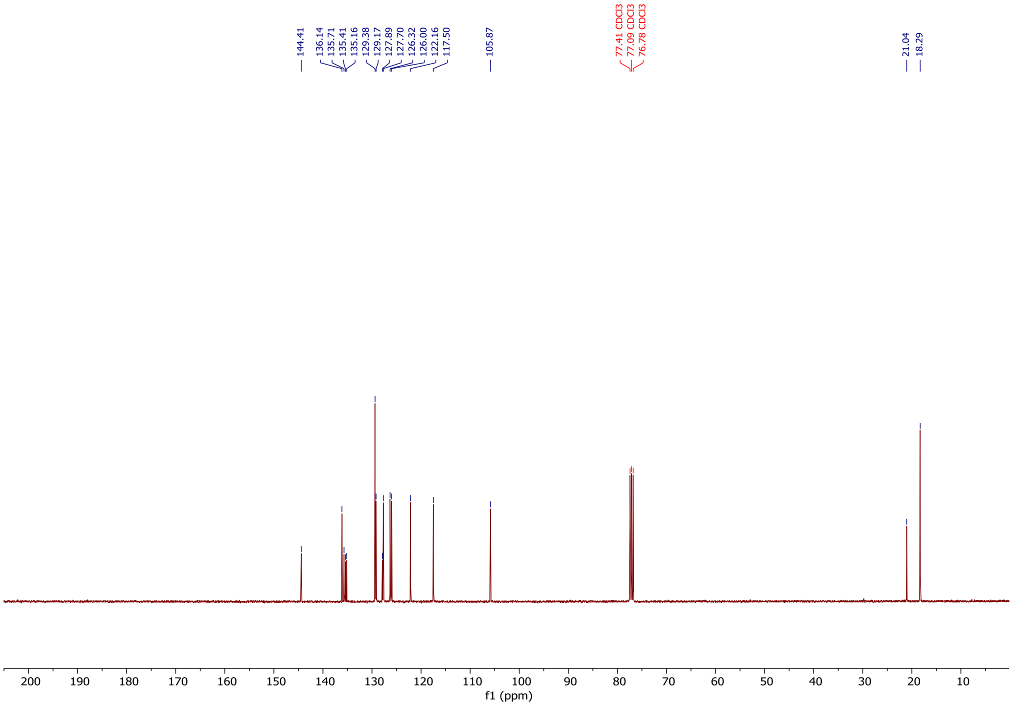

ff. *N-(3,5-dimethoxyphenyl)naphthalen-2-amine* **3g**

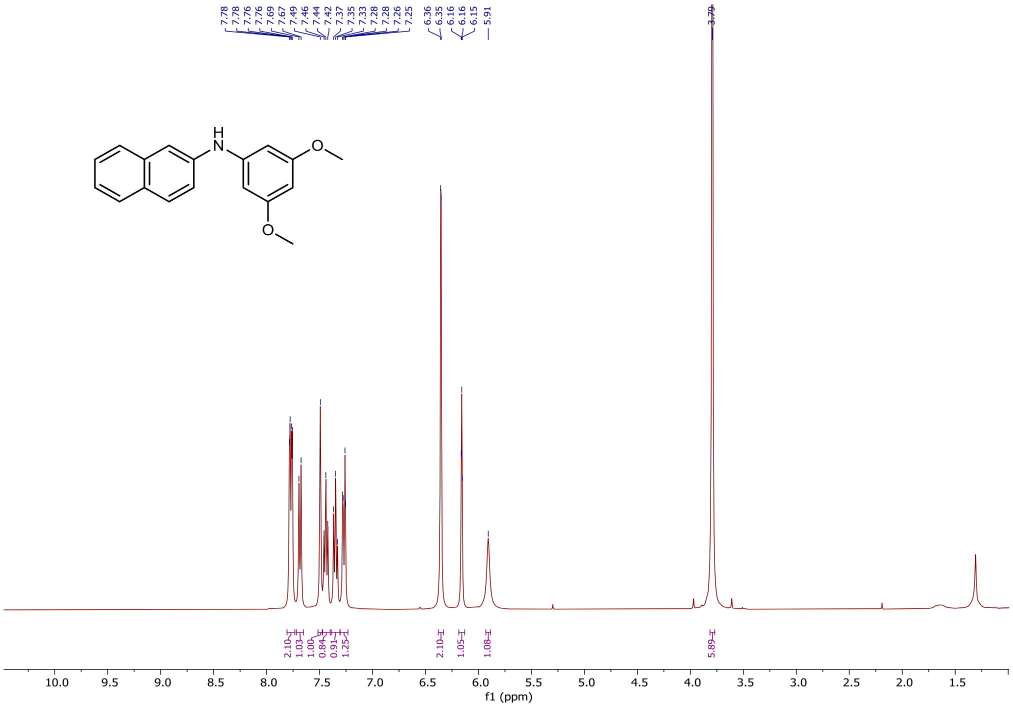

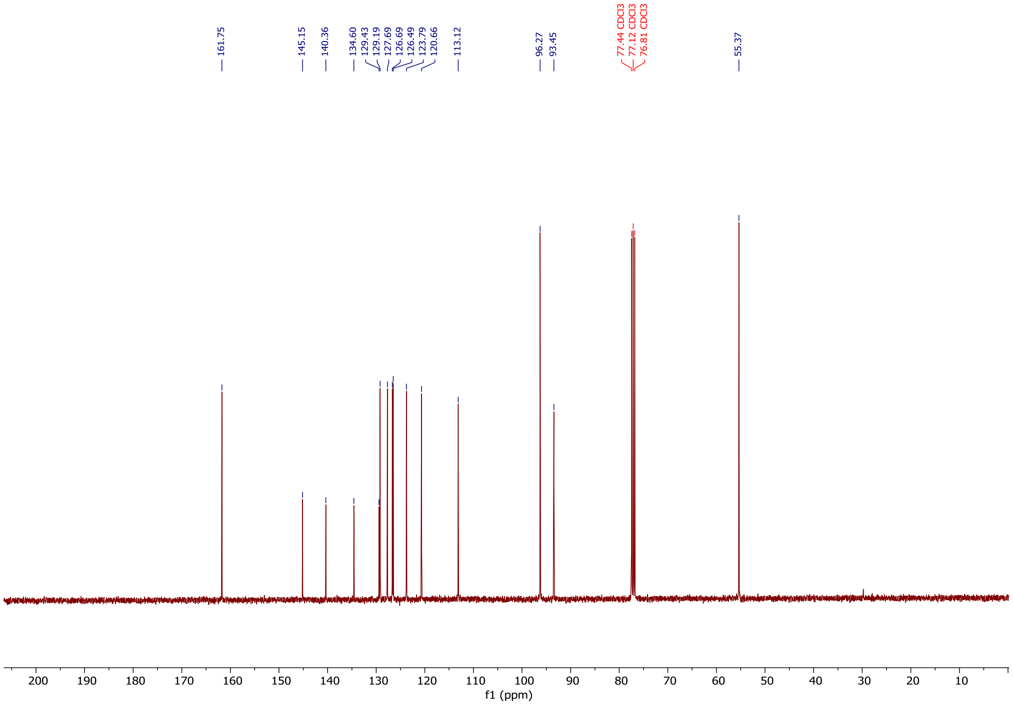

gg. *4-methyl-N-(pyridin-3-yl)pyridin-2-amine* **3h**

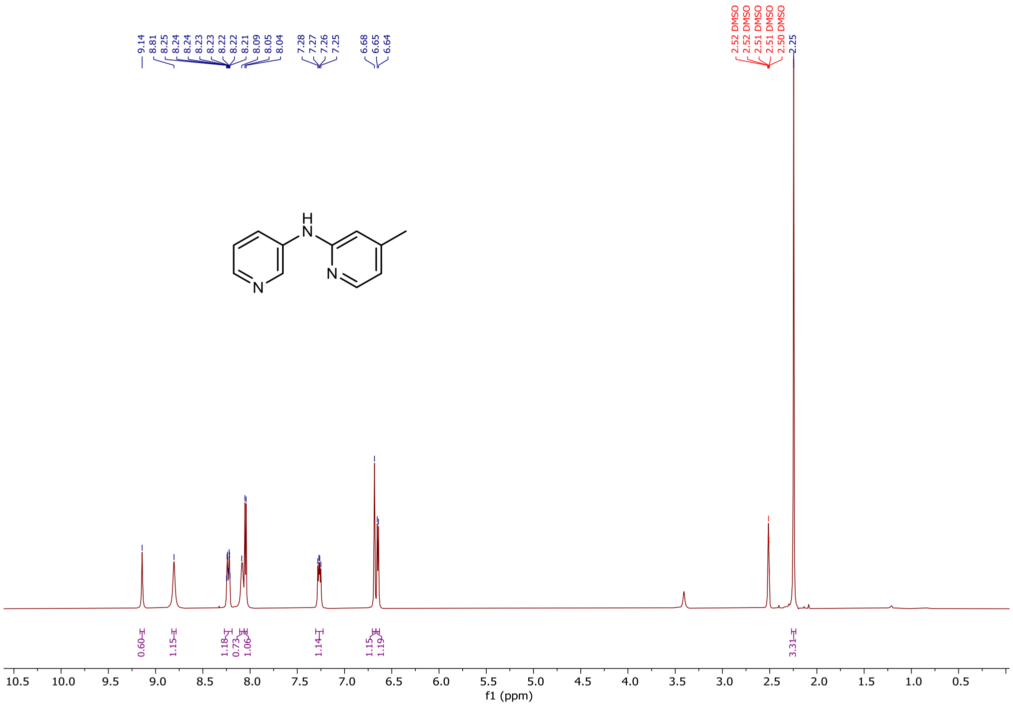

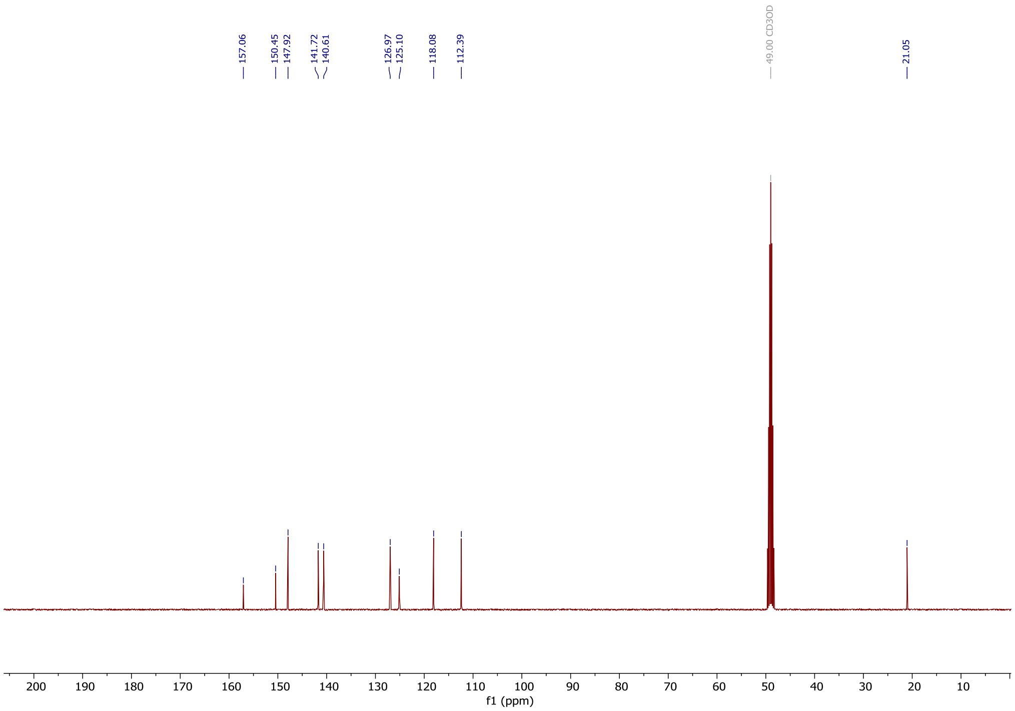

hh. *N-(6-methoxynaphthalen-2-yl)-4-methylpyridin-2-amine* **3i**

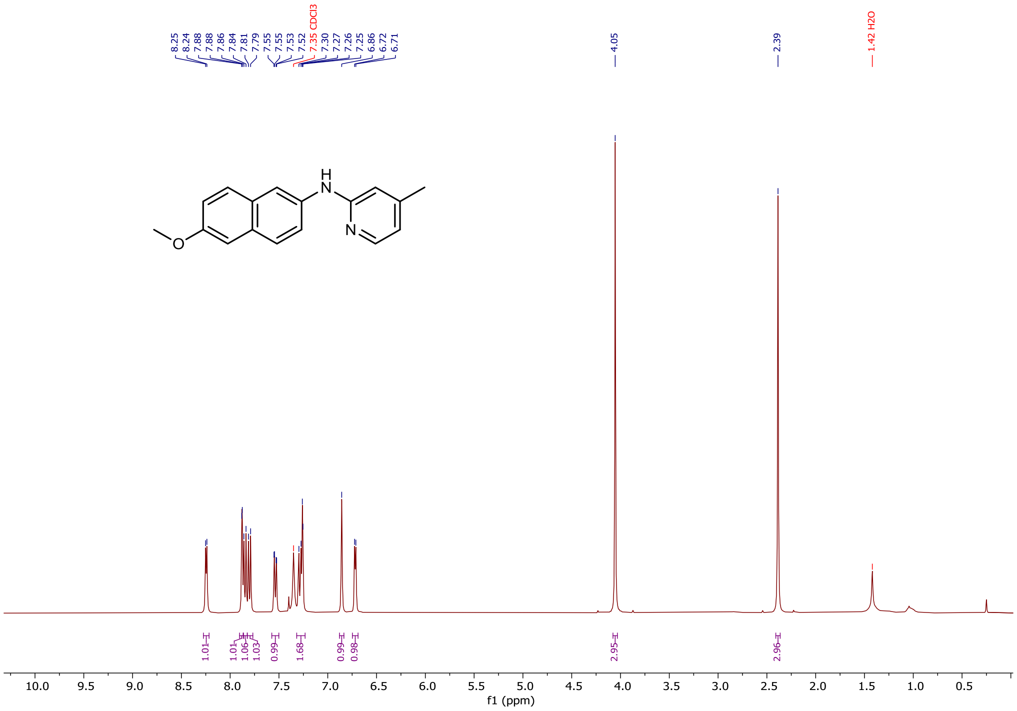

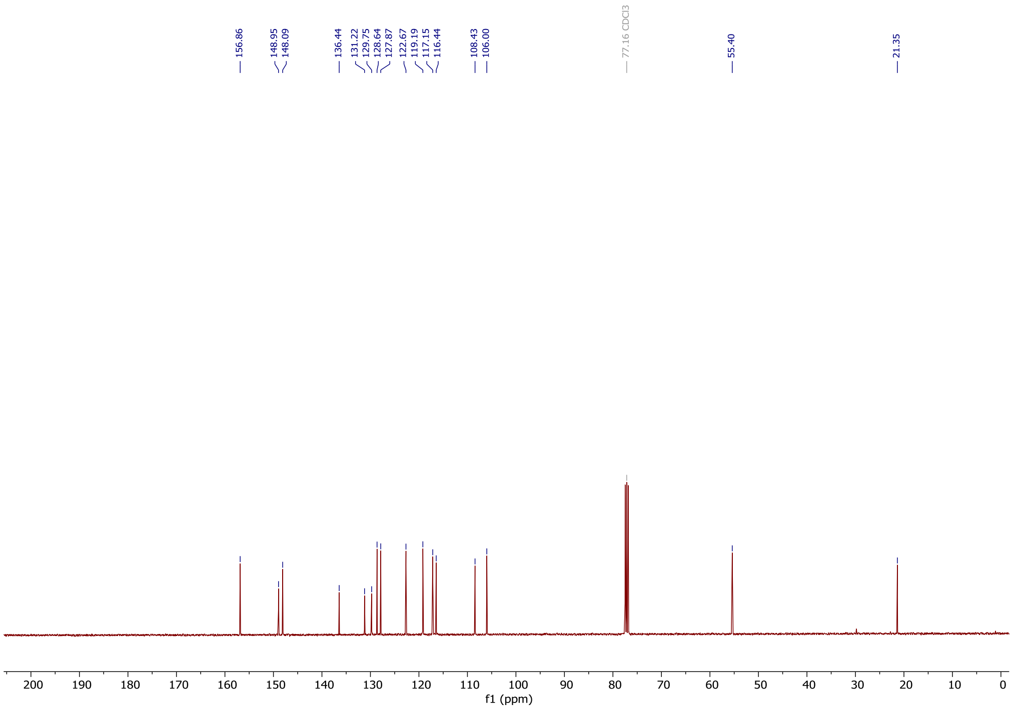

ii. *4-methyl-N-(3-(trifluoromethyl)phenyl)pyridin-2-amine* **3j**

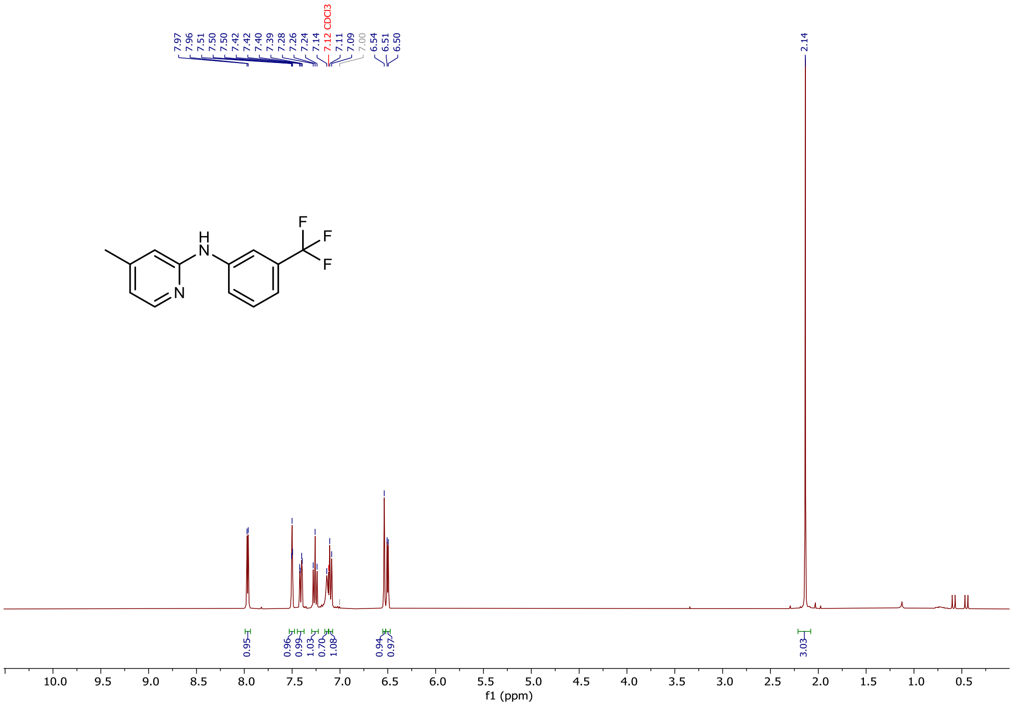

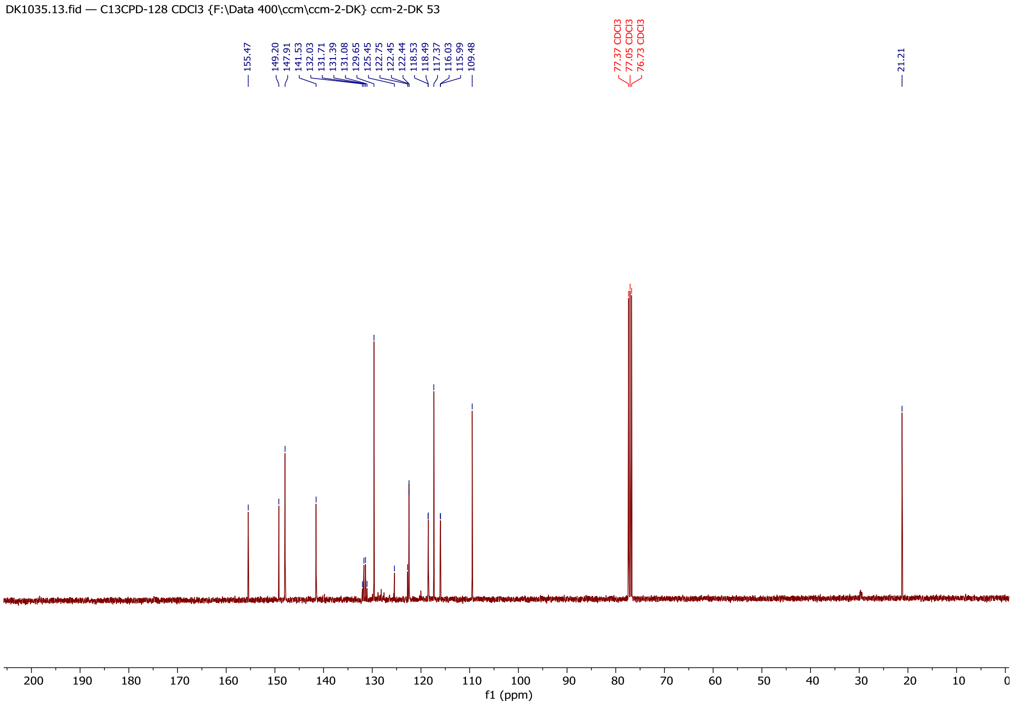

jj. *3-(naphthalen-2-ylamino)benzonitrile* **3k**

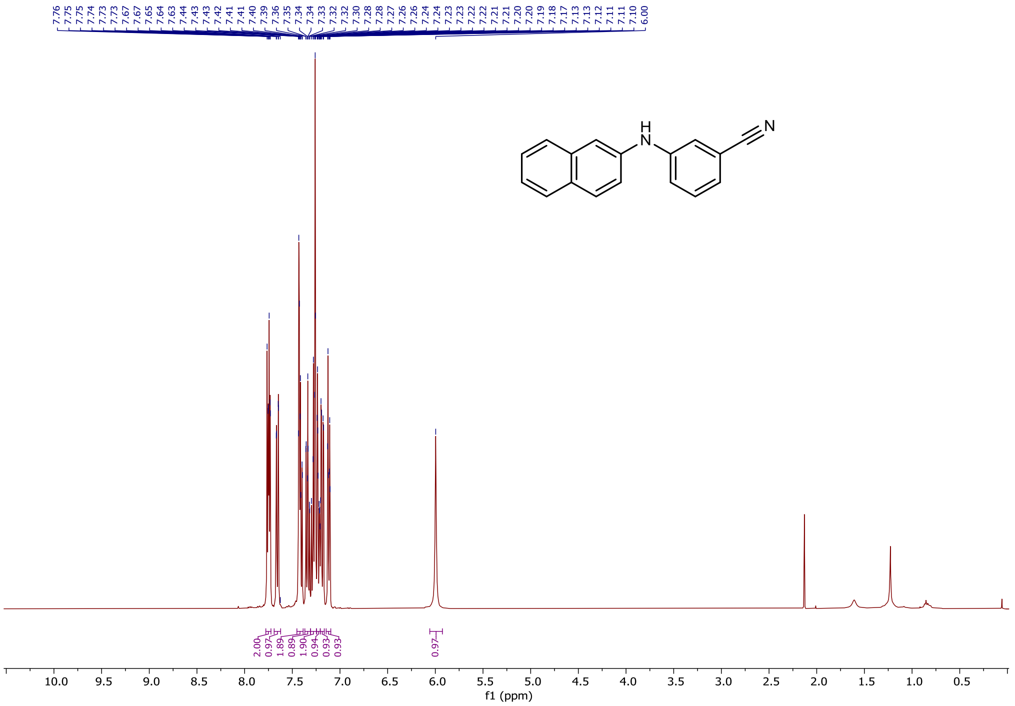

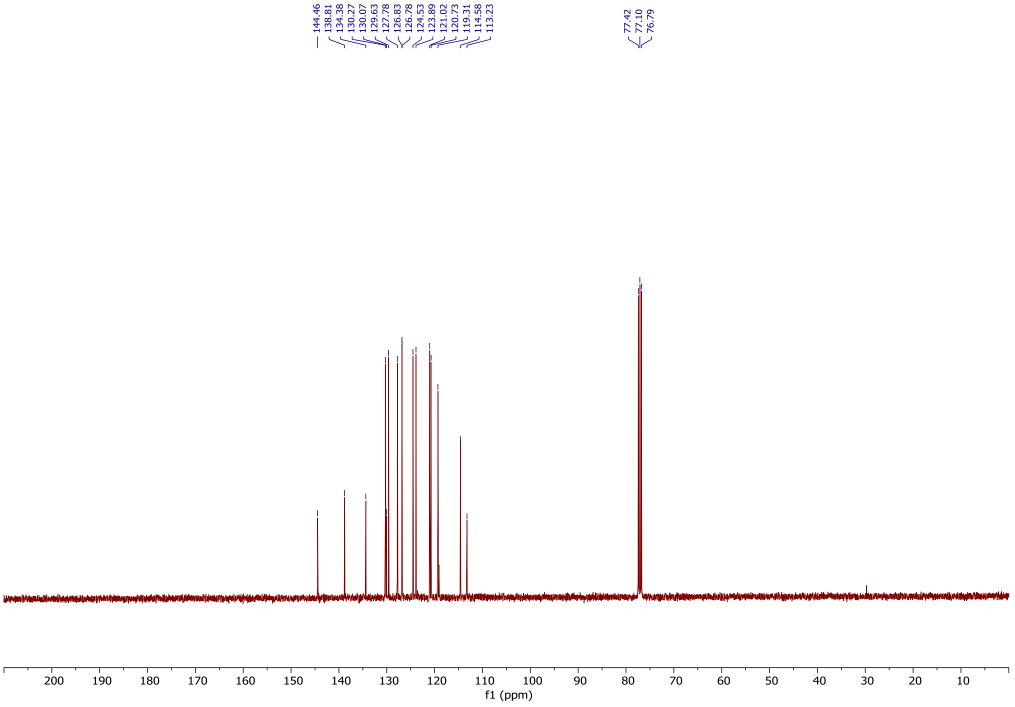

kk. *N-(4-nitrophenyl)naphthalen-2-amine* **3l**

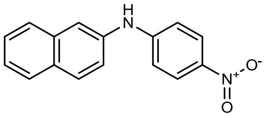

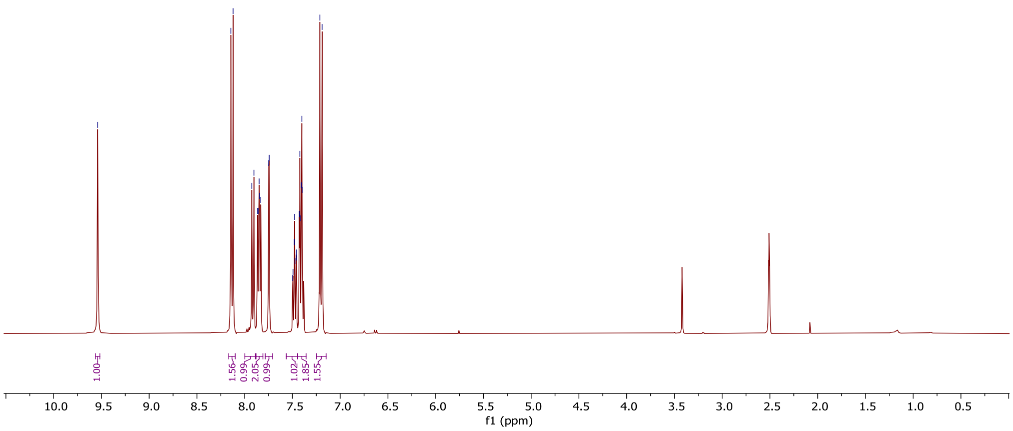

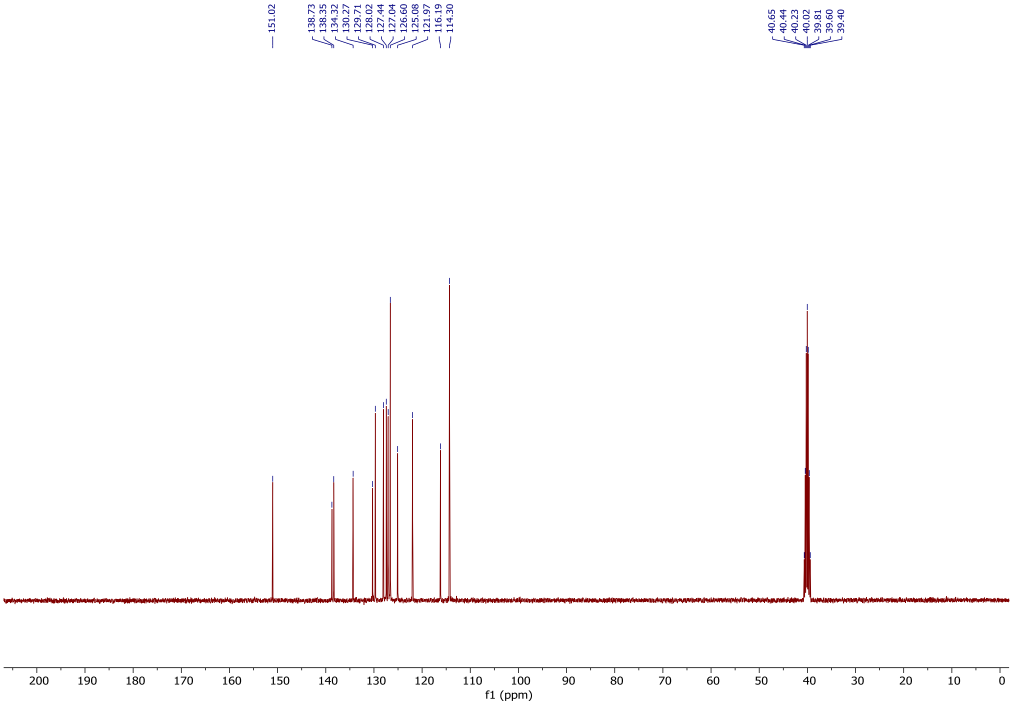

ll. *N-(3-(trifluoromethyl)phenyl)naphthalen-2-amine* **3m**

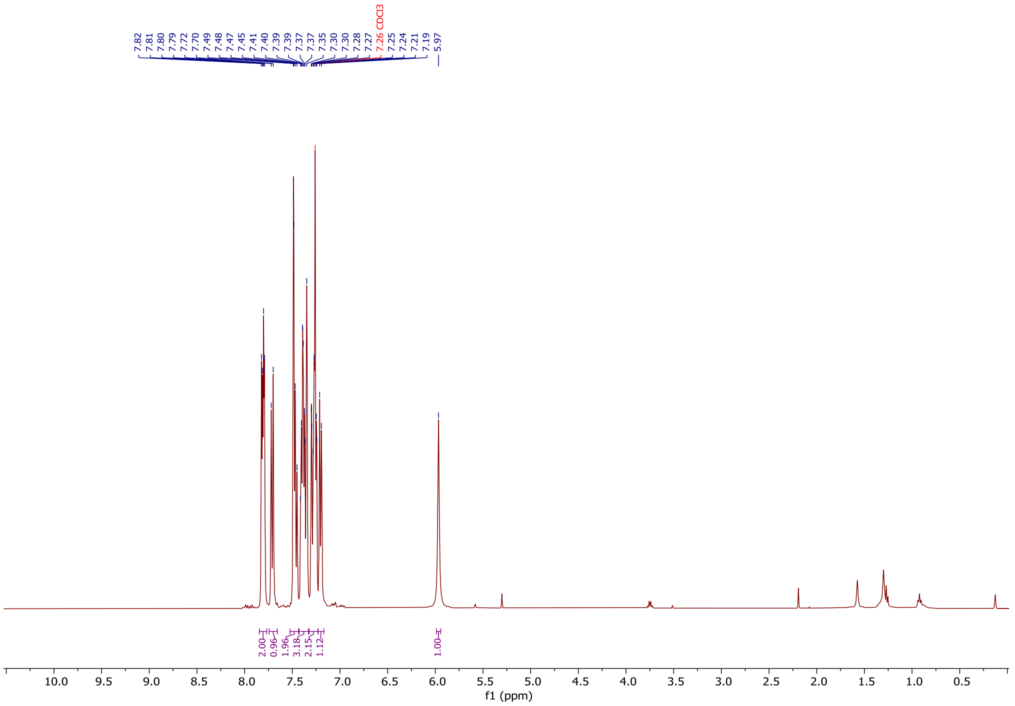

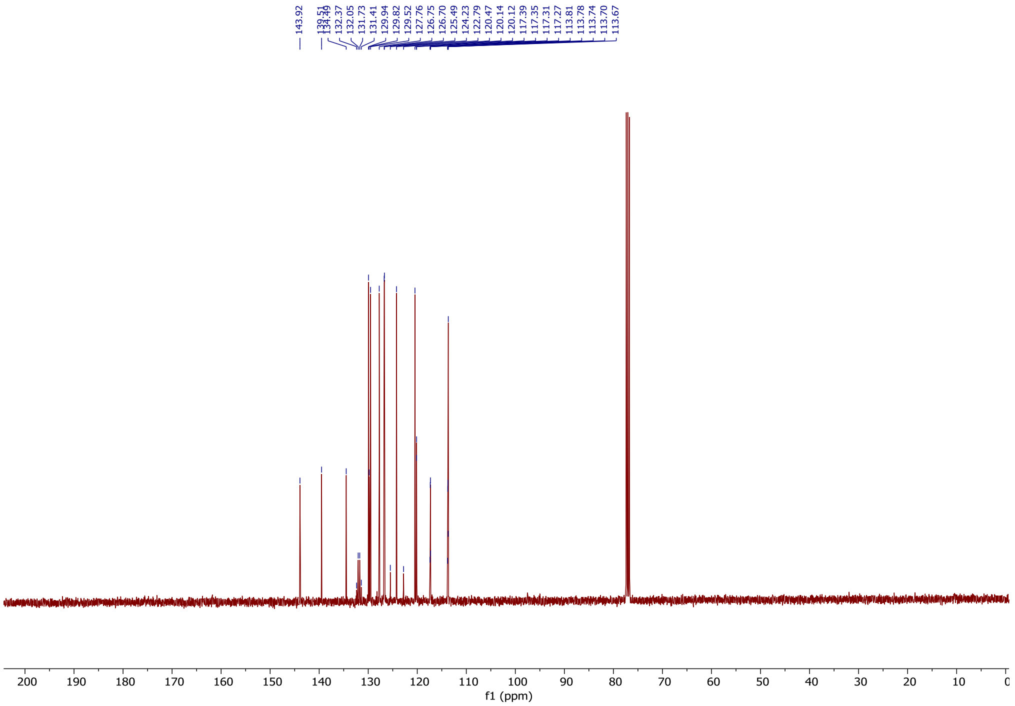

mm. *N-(4-methylpyridin-2-yl)quinolin-2-amine* **3p**

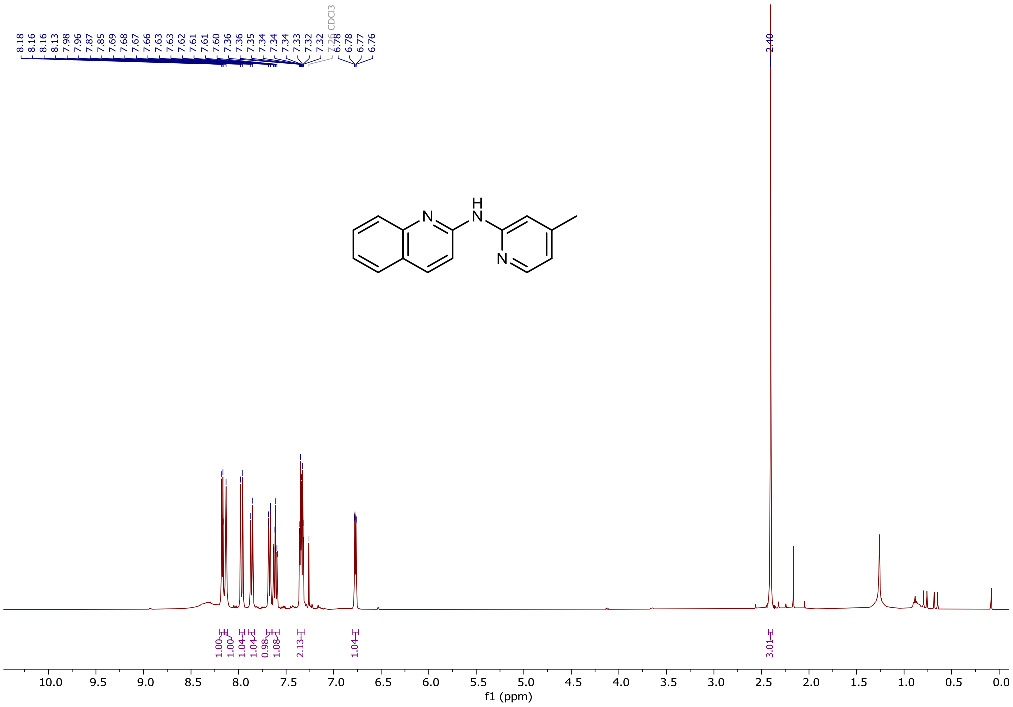

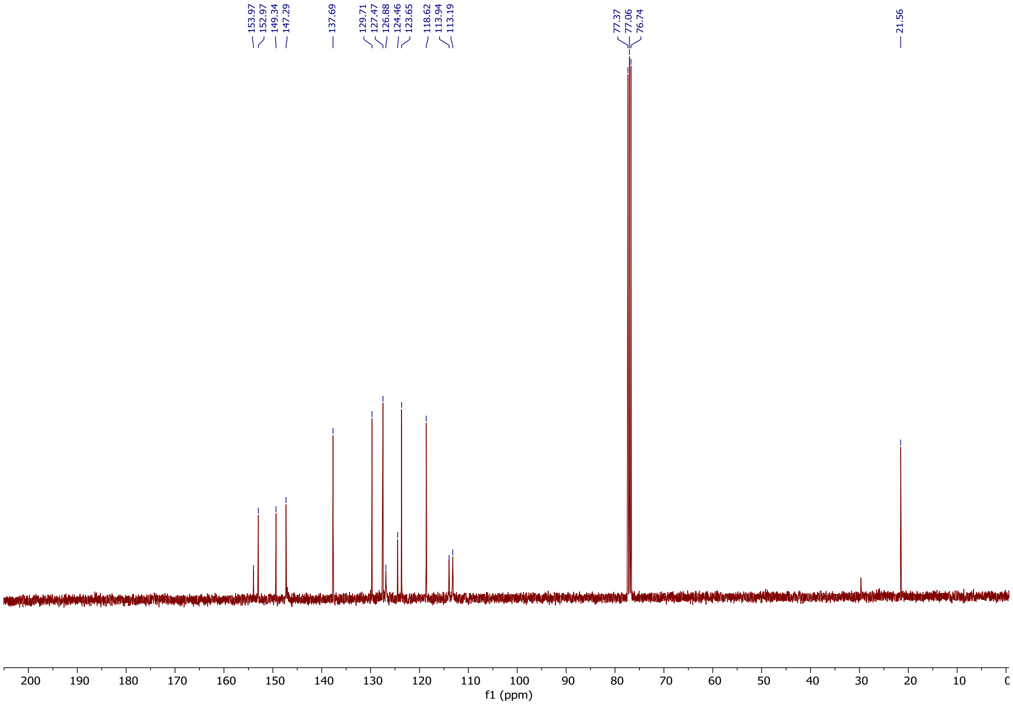

nn. *N-(naphthalen-2-yl)naphthalen-1-amine* **3q**

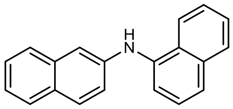

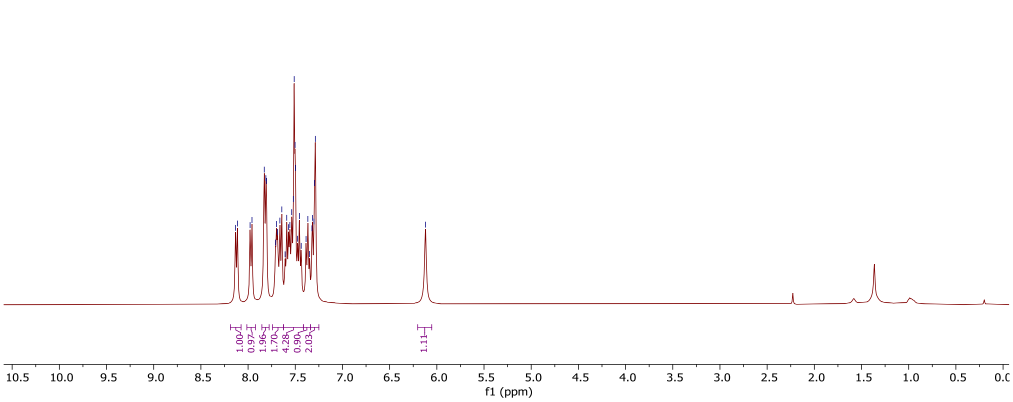

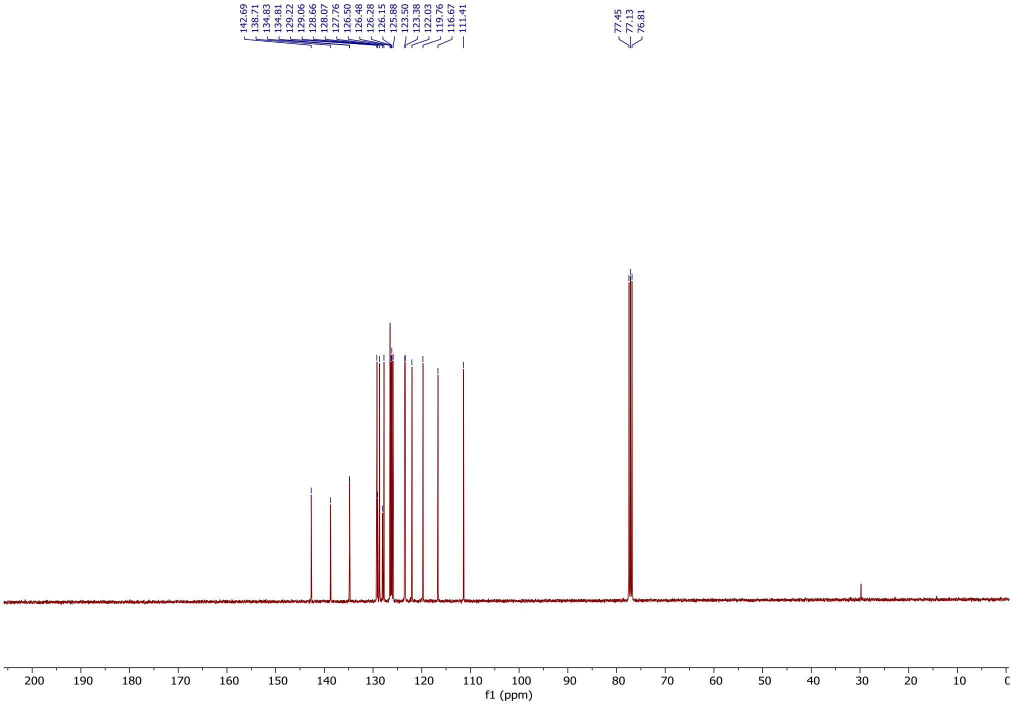

oo. *N-(naphthalen-2-yl)pyrimidin-2-amine* **3r**

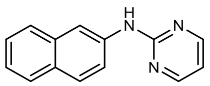

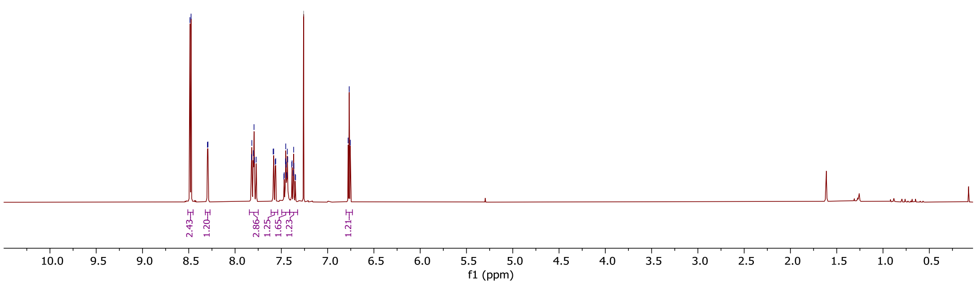

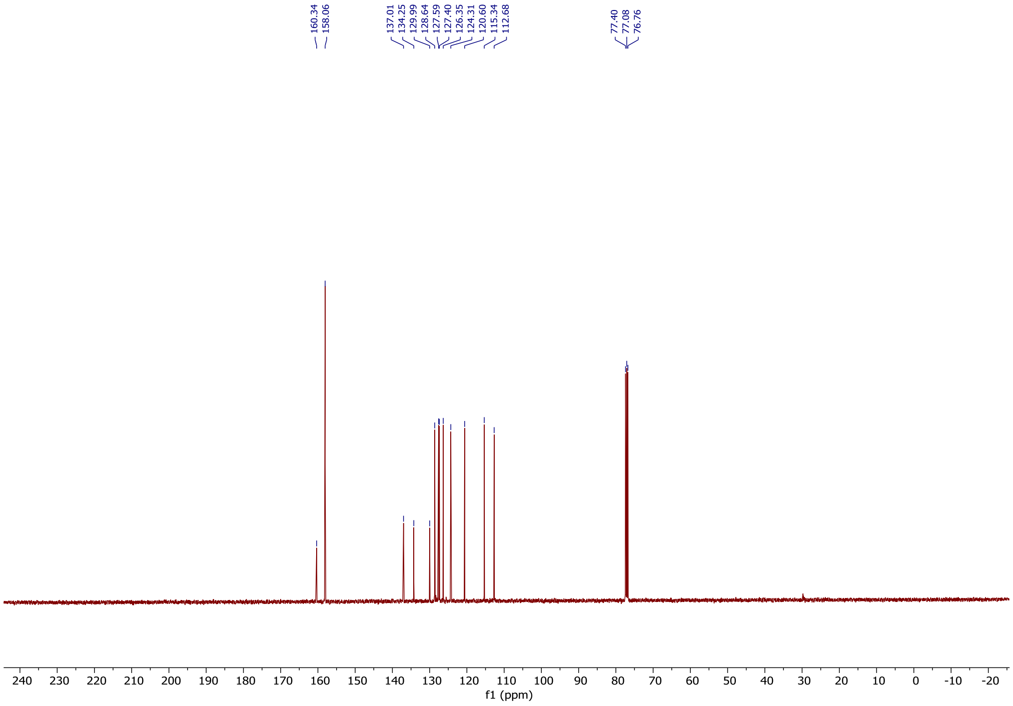

pp. *N-methyl-N-phenylnaphthalen-2-amine* **3s**

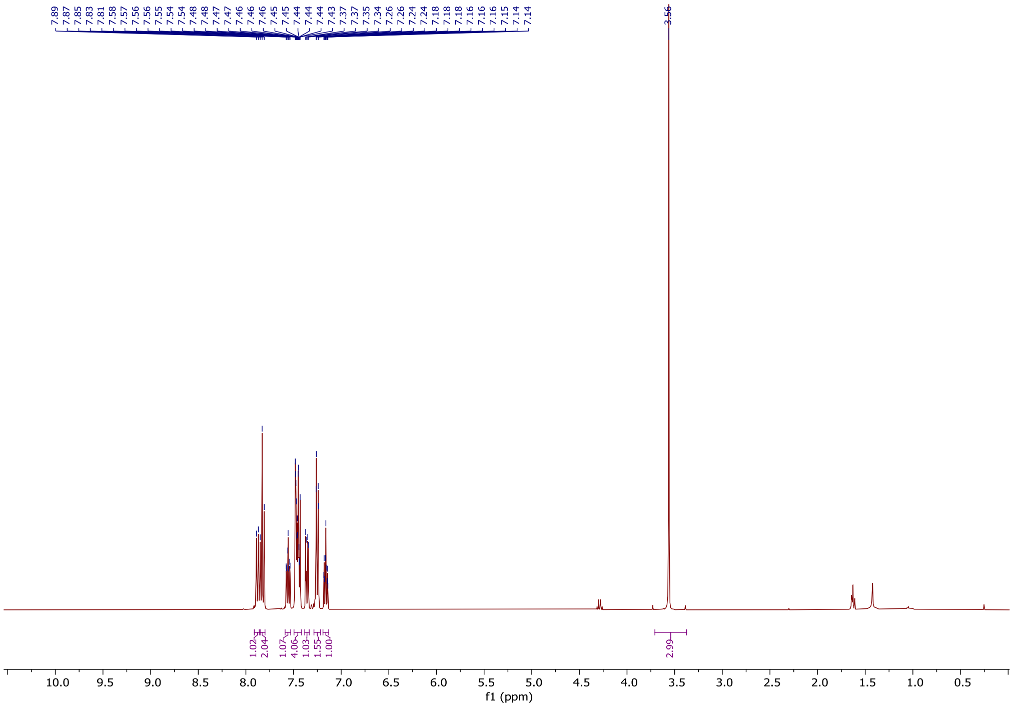

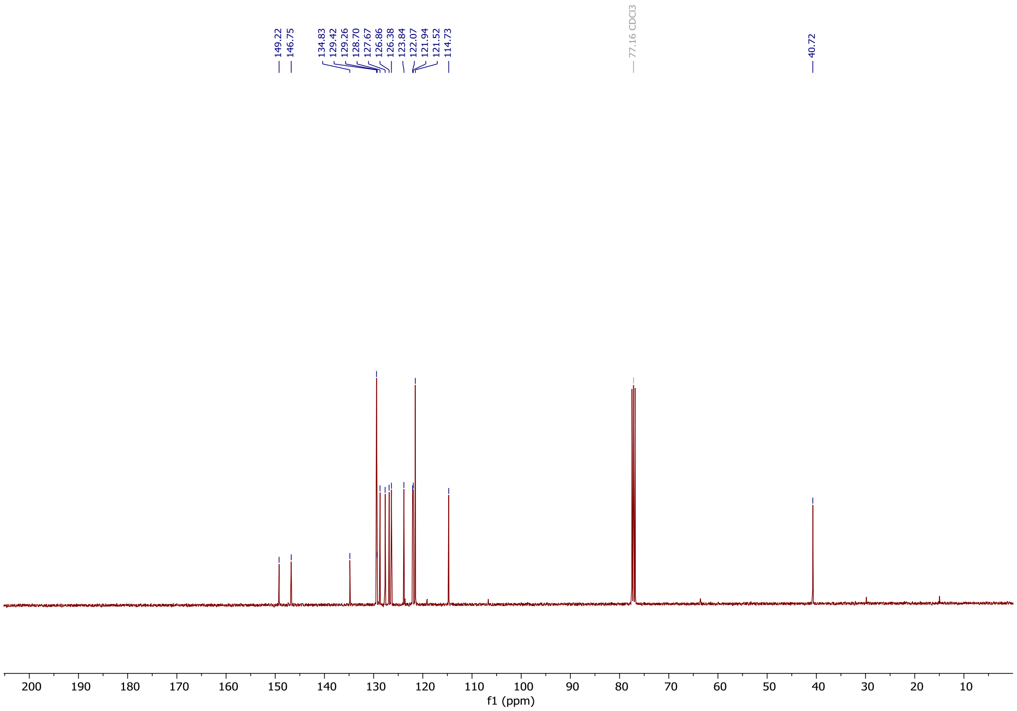

qq. *N-benzylnaphthalen-2-amine* **3t**

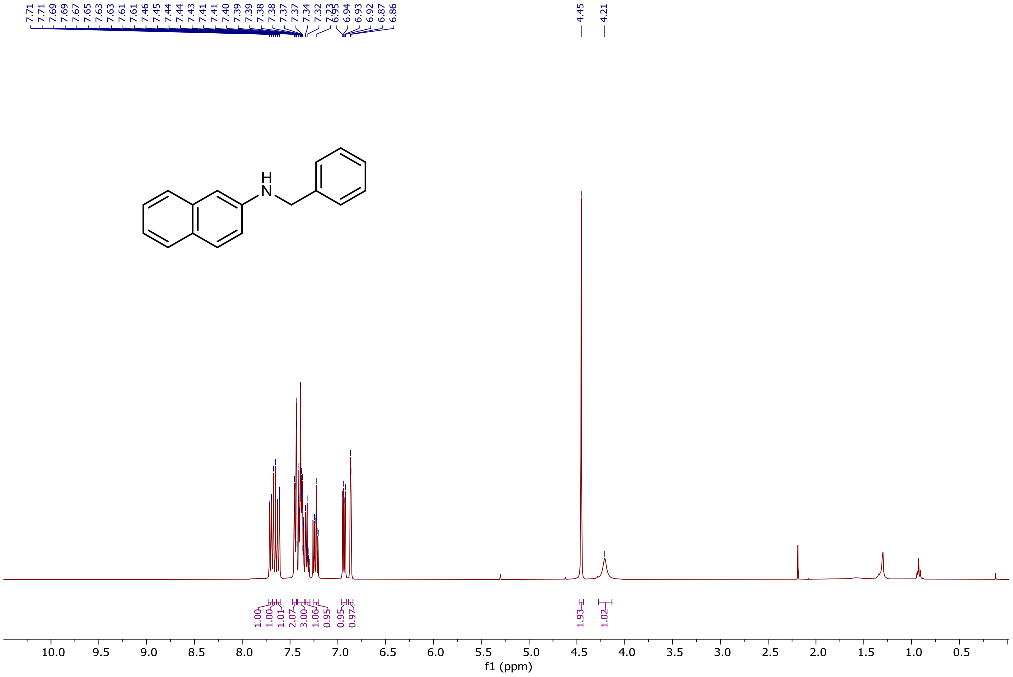

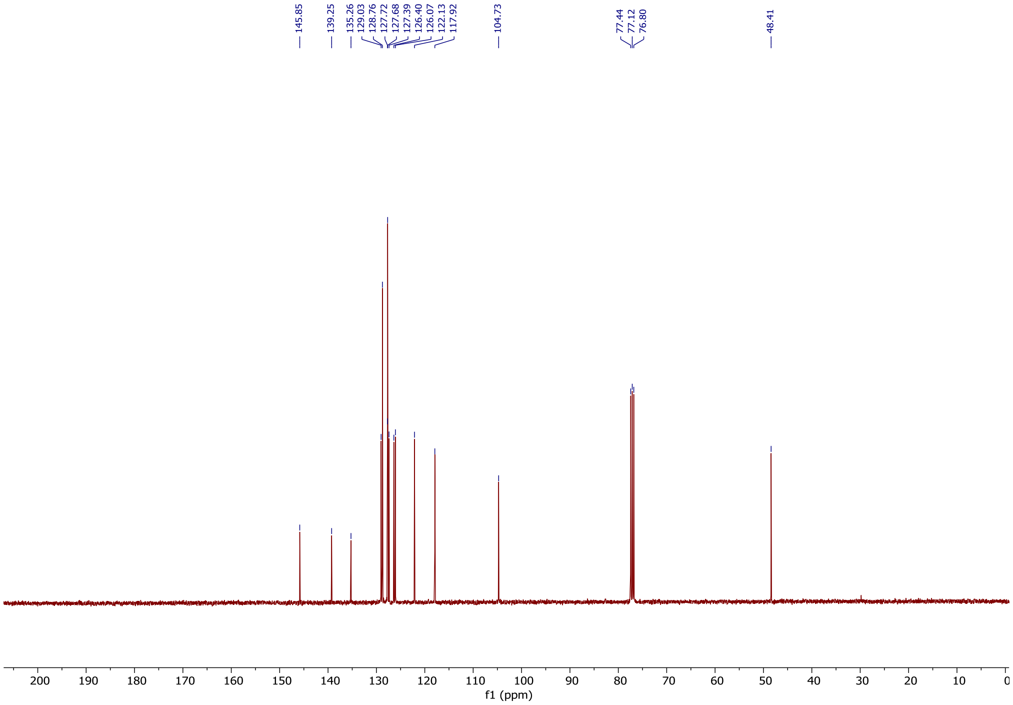

rr. *N-phenethylnaphthalen-2-amine* **3u**

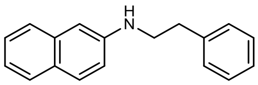

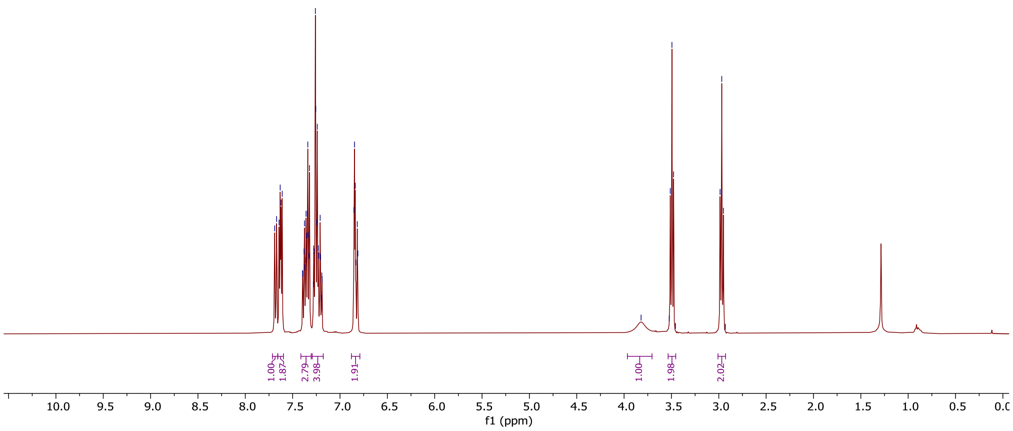

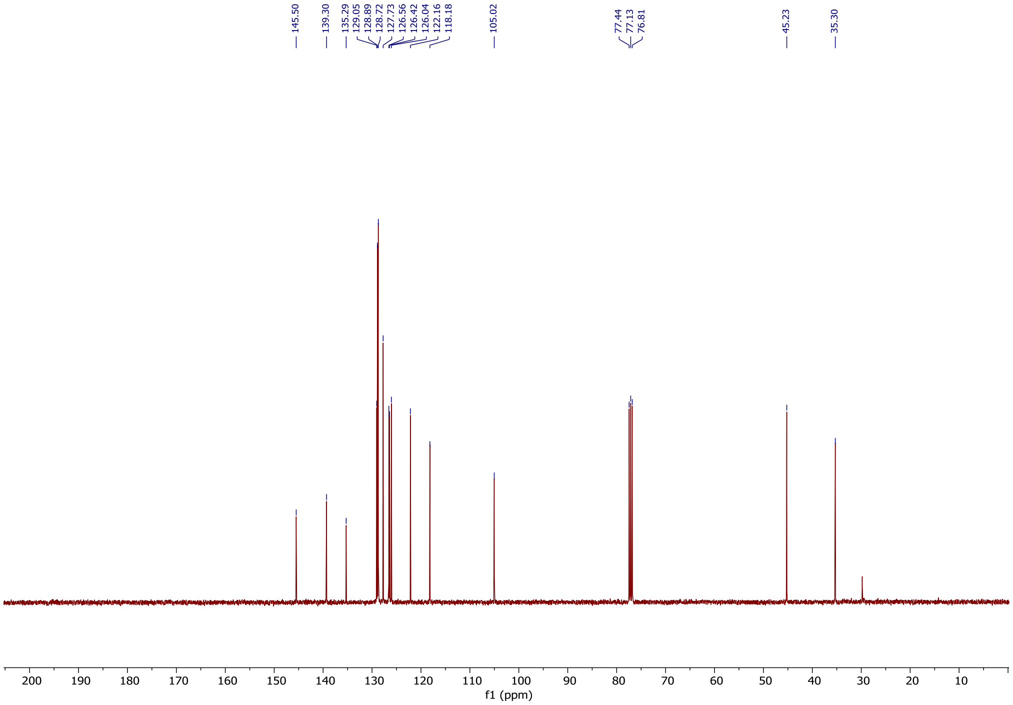

ss. *N-(naphthalen-2-yl)adamantan-1-amine* **3v**

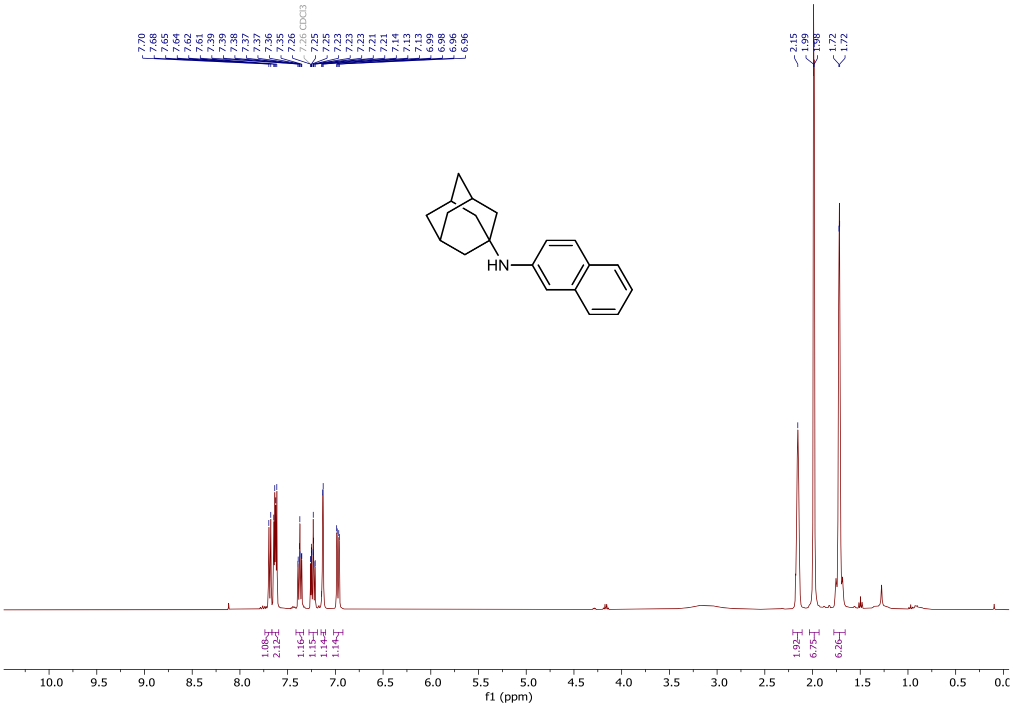

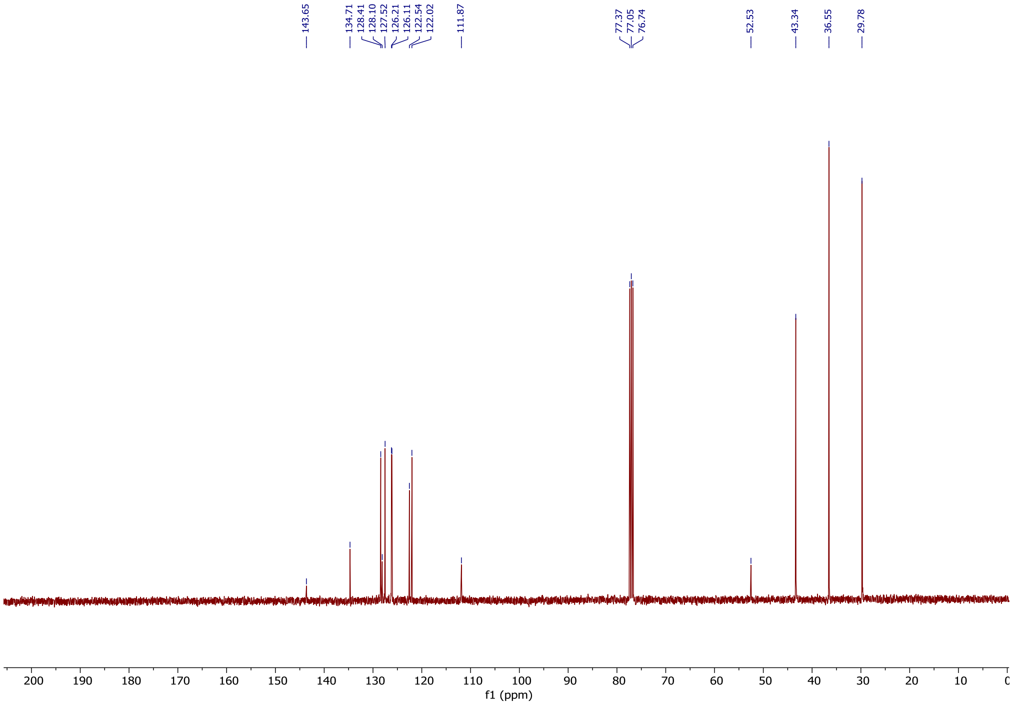

tt. *1-(naphthalen-2-yl)piperidine* **3w**

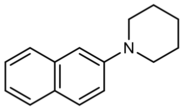

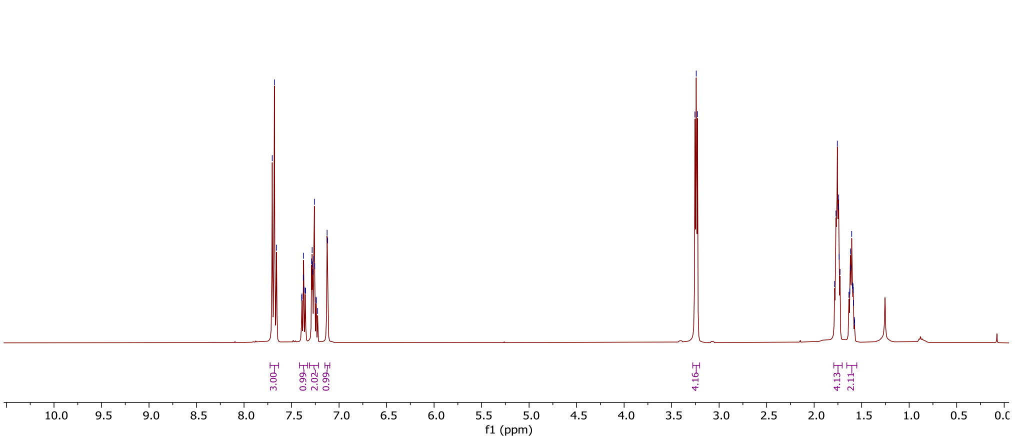

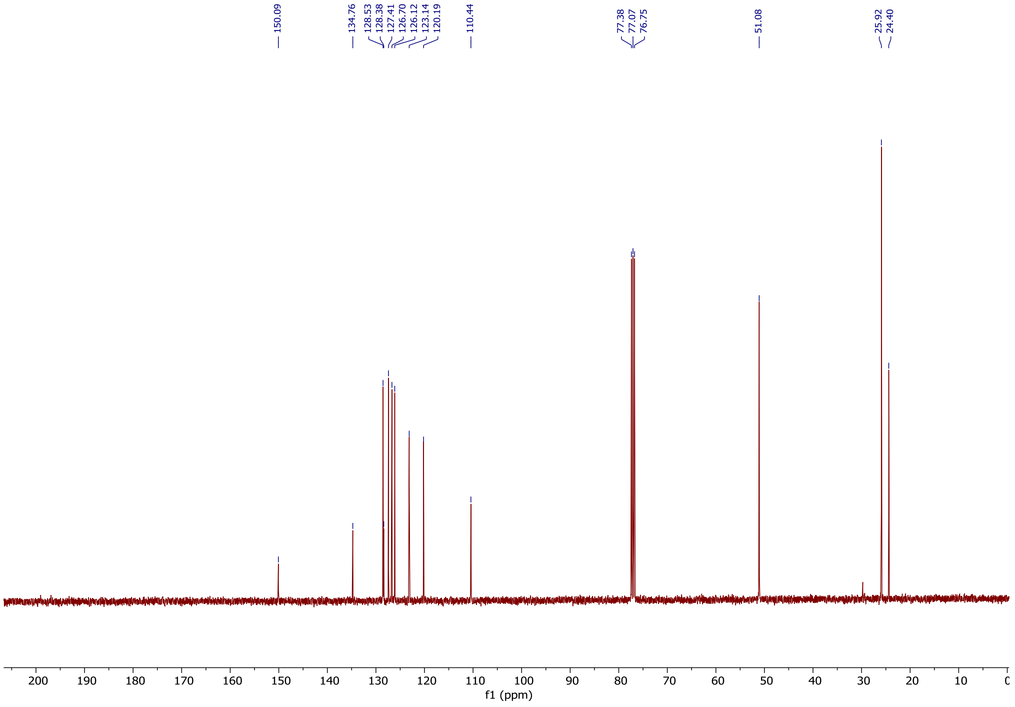

uu. *1-benzyl-4-(naphthalen-2-yl)piperazine* **3x**

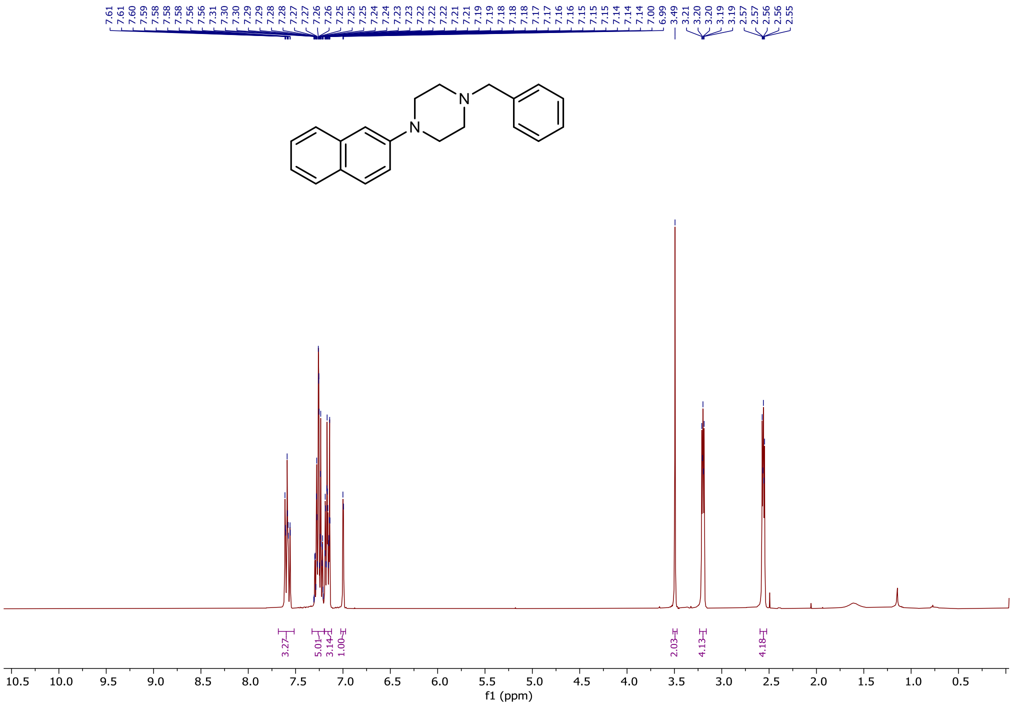

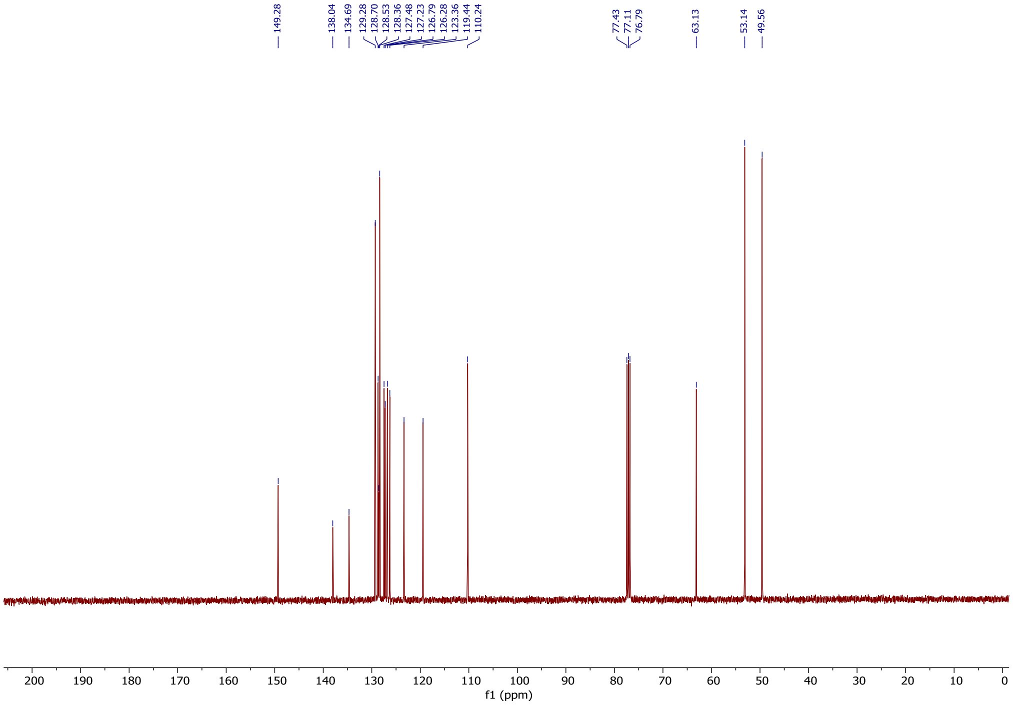

vv. *tert-butyl 4-(naphthalen-2-yl)piperazine-1-carboxylate* **3y**

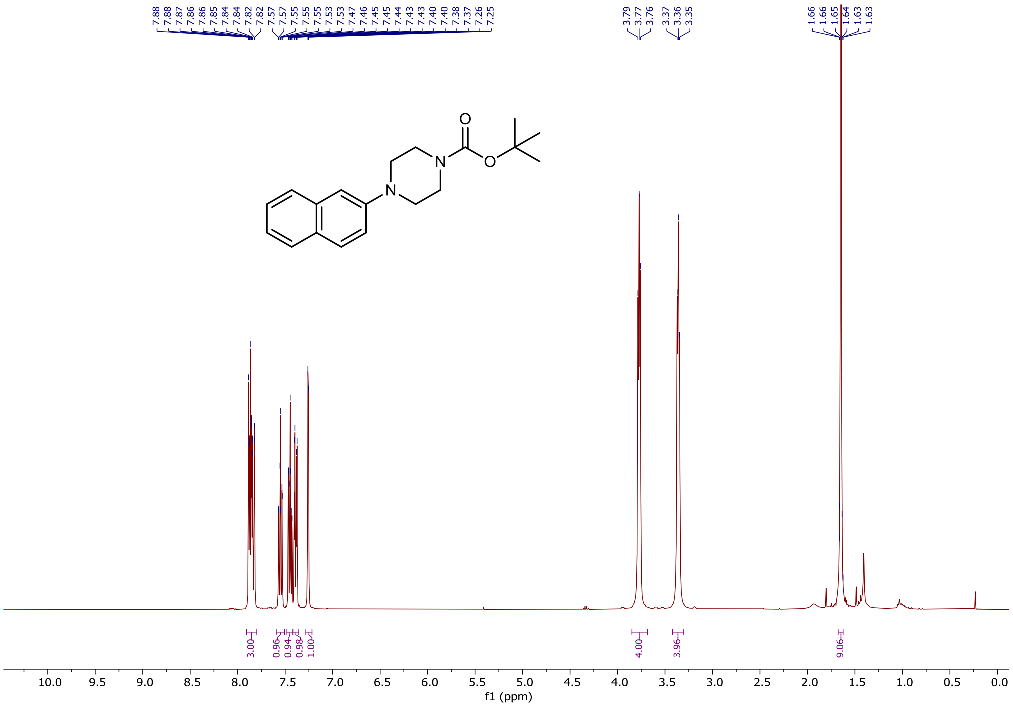

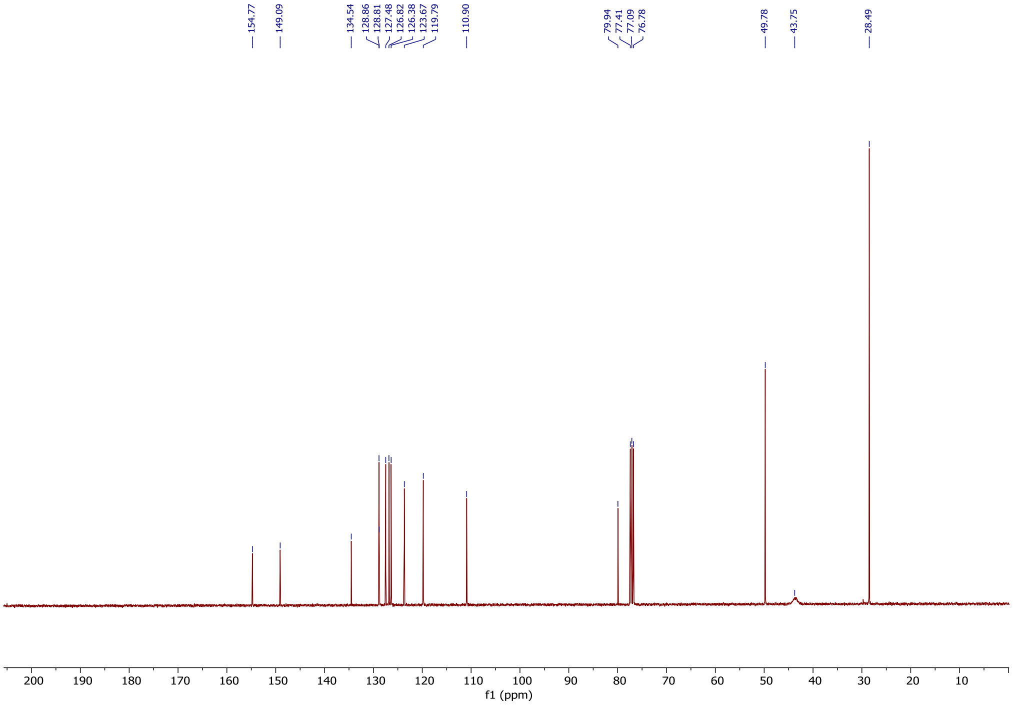

ww. *tert-butyl naphthalen-2-ylcarbamate* **3z**

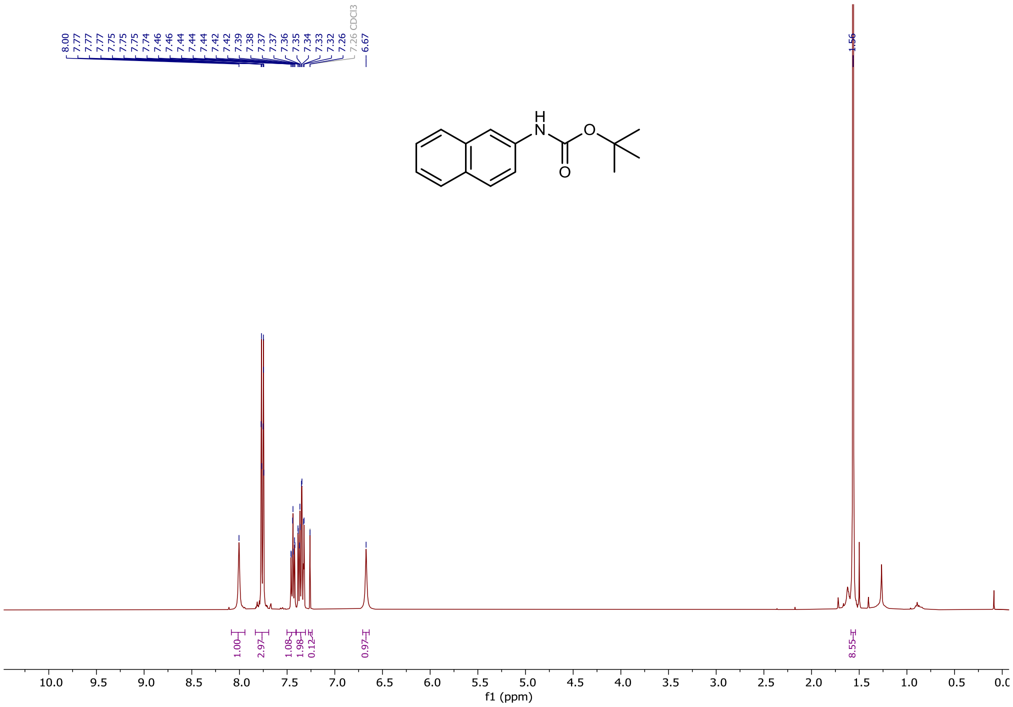

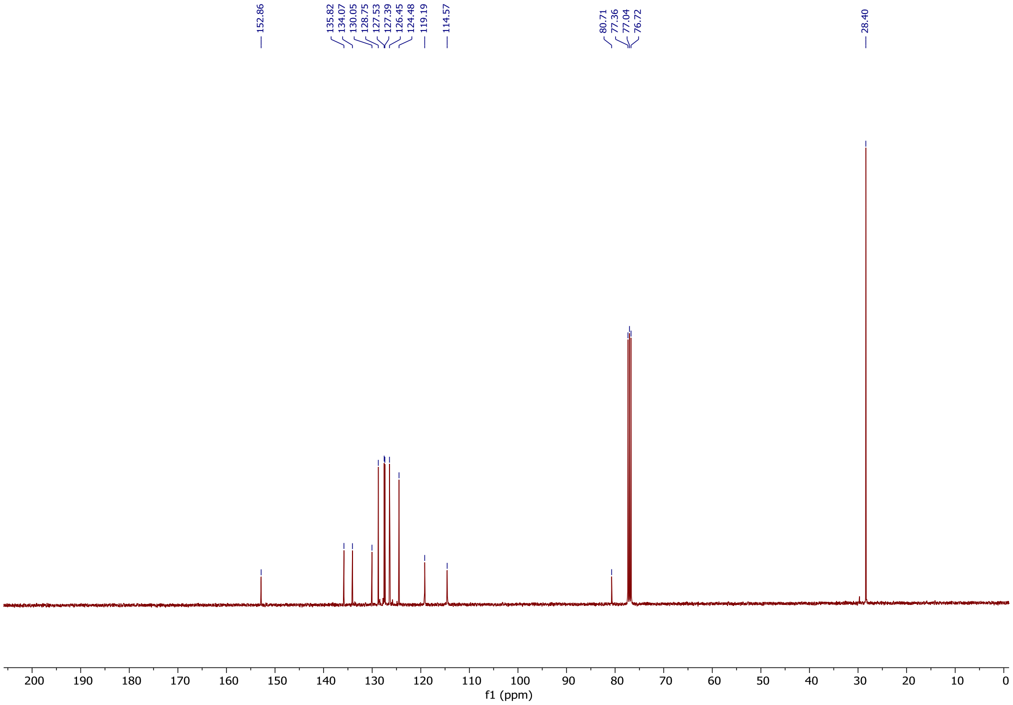

xx. *1,1-dimethyl-3-(naphthalen-2-yl)urea* **3aa**

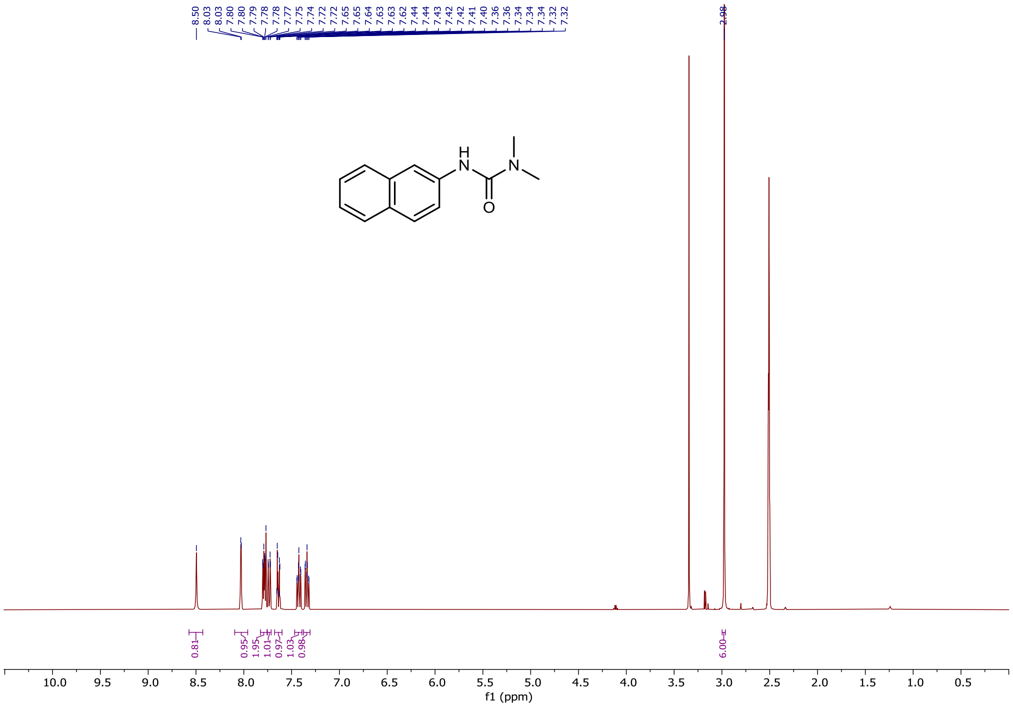

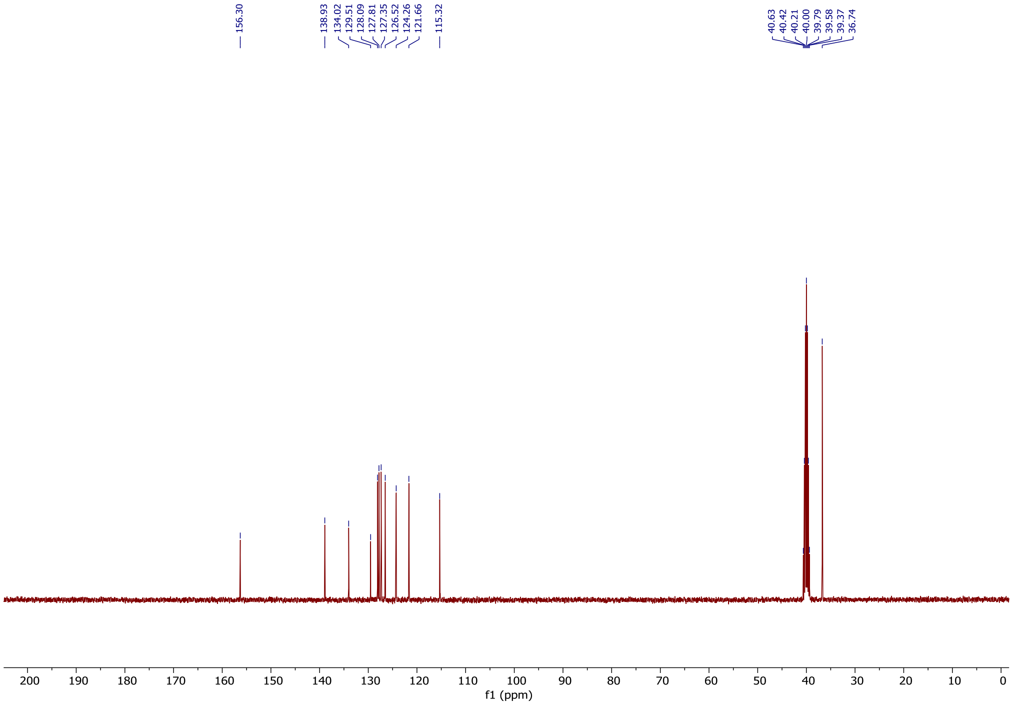

yy. *N-(naphthalen-2-yl)benzamide* **3ab**

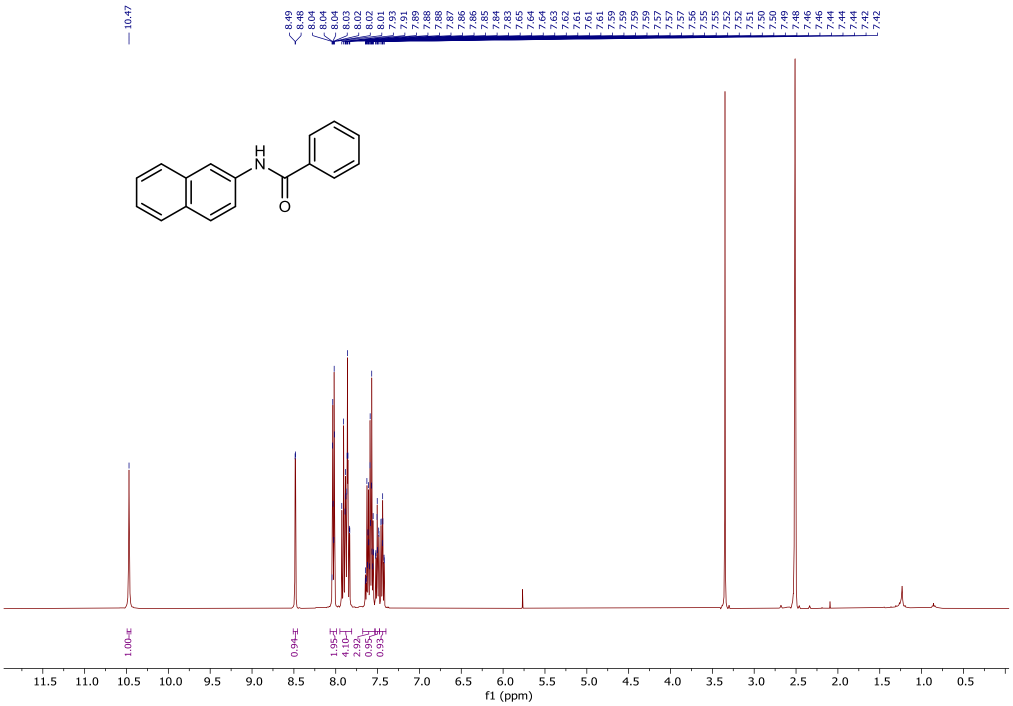

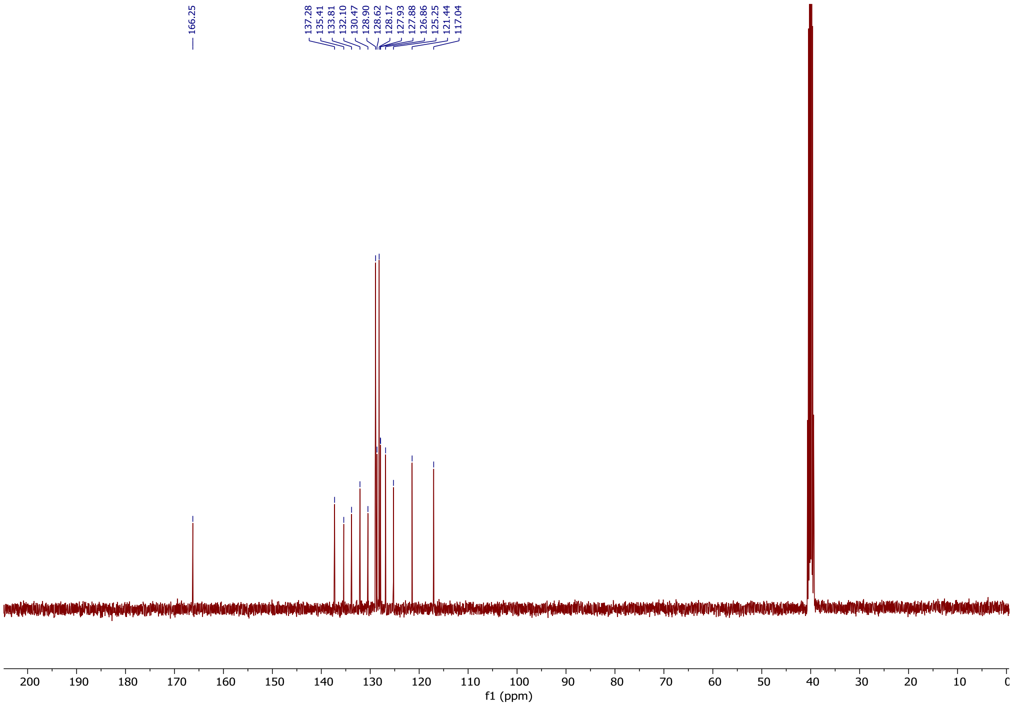

zz. *4-methoxy-N-(naphthalen-2-yl)benzamide* **3ac**

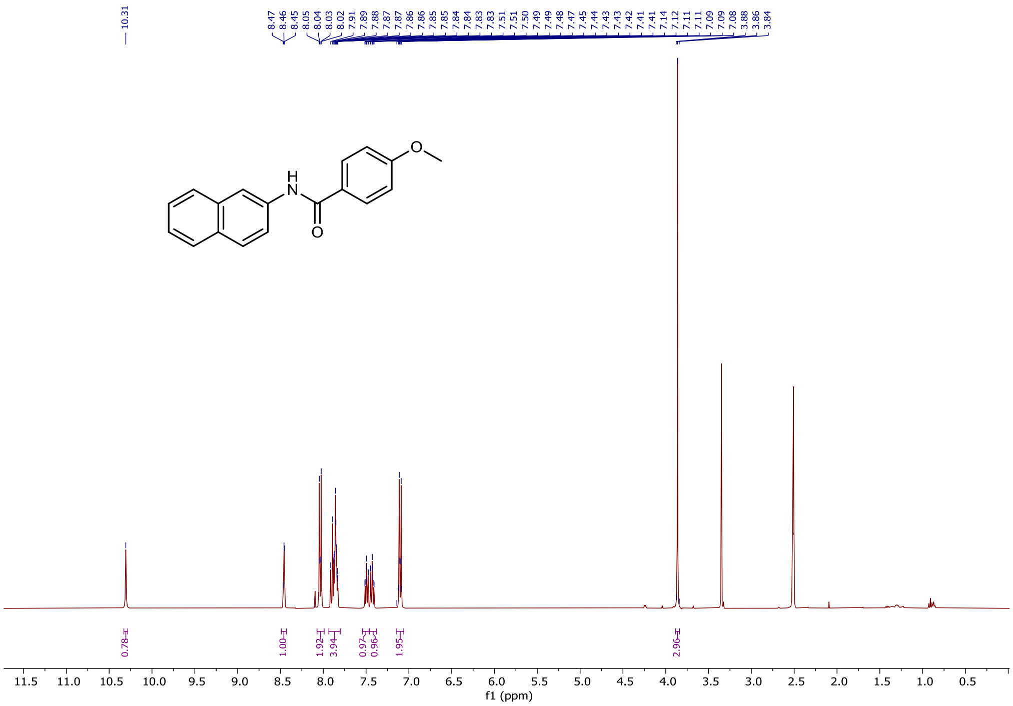

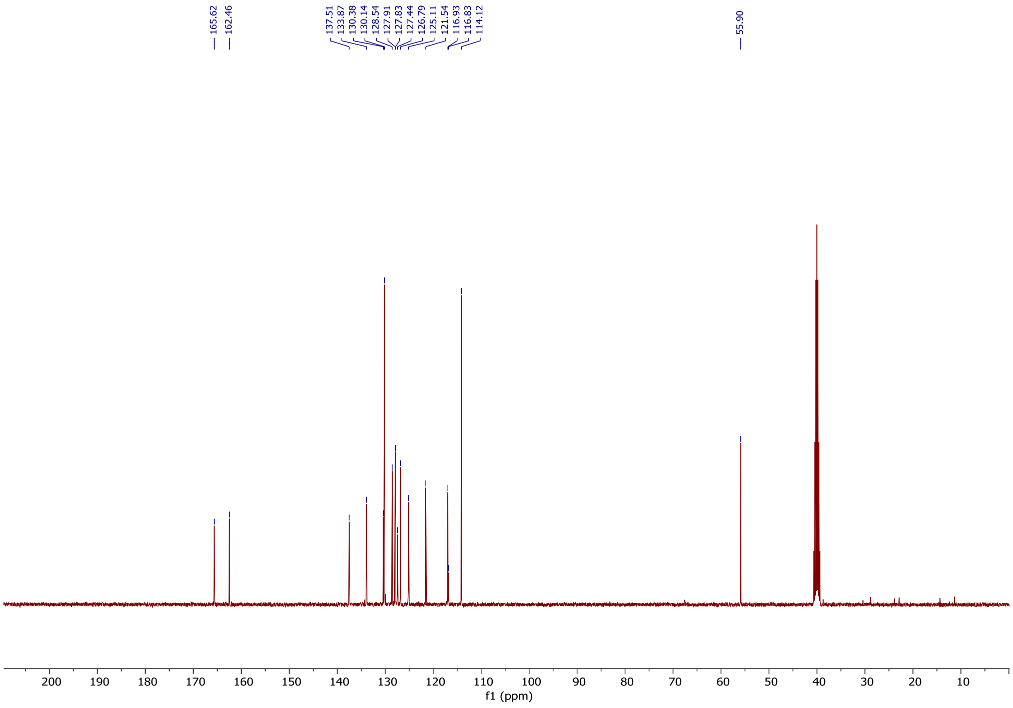

Supplement: Supplementary file 1 — Supplementary Material [file CSSC-18-e202500545-s001.pdf]
